# Intensity-dependent microcurrent stimulation enhances BMSC-derived cartilage microtissue formation under defined culture conditions

**DOI:** 10.1016/j.mtbio.2026.103698

**Published:** 2026-09-19

**Authors:** Xiang Fei, Hao Wu, Mengxing Li, Yong Xu, Hongtao He, Zhao An

**Affiliations:** a Department of Thoracic Surgery, Shanghai Pulmonary Hospital, School of Medicine, Tongji University, Shanghai, China; b Department of Clinical Laboratory, Shanghai Municipal Hospital of Traditional Chinese Medicine, Shanghai University of Traditional Chinese Medicine, Shanghai, China; c Department of Orthopedics, the Second Hospital of Dalian Medical University, Dalian, China

**Keywords:** Microcurrent stimulation, BMSCs, Cartilage microtissues, Chondrogenic differentiation, Cartilage-like matrix formation

## Abstract

Engineering phenotypically favorable cartilage-like tissue from bone marrow mesenchymal stem cells (BMSCs) remains challenging because chondrogenic differentiation can be accompanied by insufficient cartilage-associated extracellular matrix accumulation and unfavorable hypertrophic and catabolic responses. Here, we established a microcurrent-assisted culture system for scaffold-free BMSC-derived cartilage microtissues (CMTs) and investigated the intensity-dependent effects of continuous unidirectional direct-current stimulation at 0, 50, 100, 150, 250, or 500 μA under a defined electrical and culture configuration. Among the tested conditions, 150 μA (nominal electrode current density, 3 μA/mm^2^) showed the most consistently favorable overall response, with enhanced cartilage-associated gene and protein expression, greater type II collagen- and glycosaminoglycan-rich matrix accumulation, improved CMT morphology, and higher apparent mechanical properties. In contrast, 250 and 500 μA were associated with reduced cartilage-associated matrix formation and increased fibrotic, hypertrophic, catabolic, inflammatory, and apoptosis-related markers. The 150 μA condition was also associated with a distinct intracellular Ca^2+^ response pattern and increased NFATc1 nuclear localization; verapamil, BAPTA-AM, and cyclosporin A attenuated NFATc1 nuclear localization, supporting involvement of Ca^2+^/calcineurin/NFAT-related signaling without establishing a unique causal mechanism. After 2 weeks of xenogeneic subcutaneous implantation in nude mice, CMTs preconditioned at 150 μA exhibited greater cartilage-like matrix accumulation and higher apparent compressive properties than those preconditioned at 0 or 500 μA. These findings identify 150 μA as the best-performing tested condition under the present electrical configuration and provide short-term ectopic proof-of-concept evidence, while longer-term orthotopic studies are required to evaluate phenotypic stability, tissue integration, and functional outcomes in cartilage-defect models.

## Highlights

• Microcurrent stimulation enhanced BMSC-derived cartilage organoids.
• 150 μA was optimal for organoid maturation and matrix deposition.
• Optimized stimulation improved COL II/GAG-rich cartilage matrix.
• Ca^2+^/calcineurin/NFAT signaling mediated the pro-chondrogenic effect.
• Excessive current impaired organoid quality and cartilage regeneration.

## Graphical abstract

**Figure:**
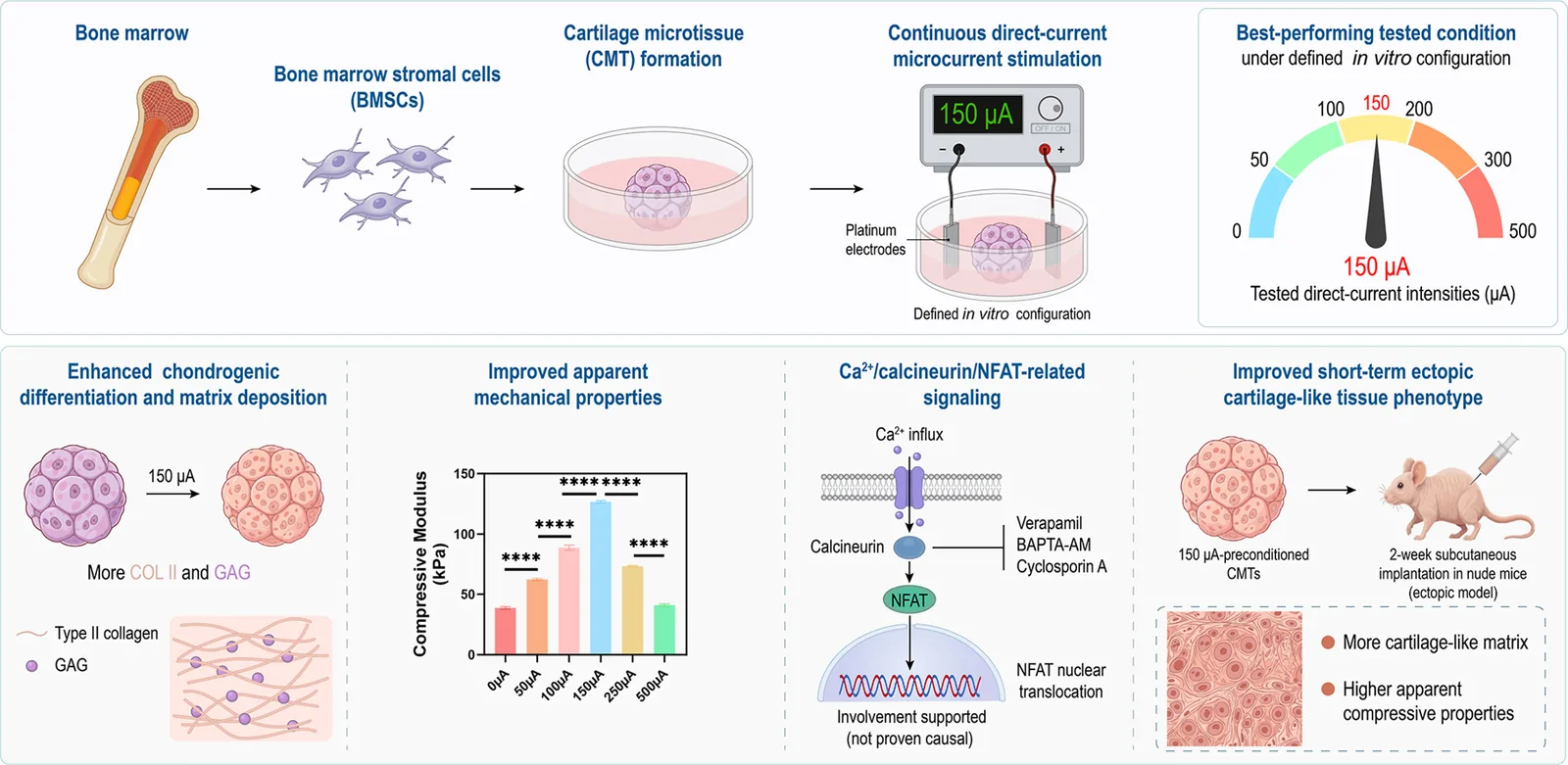

## 1 Introduction

Cartilage defects remain a major challenge in regenerative medicine because cartilage has limited intrinsic repair capacity owing to its low cellularity, restricted vascularization, and slow matrix turnover [1,2]. Existing reconstructive and regenerative approaches, including autologous grafting, alloplastic implants, microfracture-based procedures, and cell-based therapies, are limited by factors such as donor-site morbidity, restricted tissue availability, fibrocartilage formation, insufficient matrix maturation, and incomplete functional integration [3,4]. Therefore, reliable strategies for generating mature and phenotypically favorable cartilage-like tissue remain needed for musculoskeletal and reconstructive applications.

Bone marrow mesenchymal stem cells (BMSCs) are a widely investigated cell source for cartilage tissue engineering because of their expansion capacity and chondrogenic potential [5,6]. Under appropriate induction conditions, BMSCs can produce cartilage-associated extracellular matrix (ECM), including type II collagen (COL II), aggrecan, cartilage oligomeric matrix protein, and sulfated glycosaminoglycans (GAGs) [7]. However, BMSC chondrogenesis is frequently accompanied by insufficient cartilage-specific matrix accumulation, hypertrophic differentiation, matrix degradation, and stress-associated responses, which can compromise the quality of engineered constructs [8]. Three-dimensional (3D) aggregation provides a useful scaffold-free platform because high-density cell condensation enhances cell–cell interactions and facilitates local retention of secreted ECM [9,10]. In the present study, we refer to these constructs as BMSC-derived cartilage microtissues (CMTs), operationally defined as scaffold-free 3D BMSC aggregates generated under chondrogenic induction that develop cartilage-like ECM.

Bioelectrical stimulation has emerged as a nonchemical biophysical approach for modulating cell behavior and tissue formation [11,12]. Cartilage is responsive to electrical and electrochemical cues associated with ion transport, mechanotransduction, matrix metabolism, and cellular homeostasis [13]. Microcurrent stimulation is particularly attractive for tissue-engineering applications because it can provide a mild and controllable electrical input that is readily incorporated into *in vitro* culture systems [14]. Importantly, however, biological responses to electrical stimulation are strongly parameter- and configuration-dependent [15]. Insufficient stimulation may produce little biological effect, whereas excessive electrical input may disrupt cellular homeostasis and impair tissue quality [16]. Consequently, the relevant question is not whether stronger stimulation is uniformly beneficial, but whether a favorable stimulation range can be identified under a defined electrode and culture configuration.

The mechanisms by which BMSCs sense microcurrent stimulation remain incompletely understood [17]. In this study, the Ca^2+^/calcineurin/nuclear factor of activated T cells (NFAT) axis was investigated as a hypothesis-driven candidate pathway, rather than as a pathway identified by unbiased transcriptomic or proteomic screening. This rationale was based on evidence that intracellular Ca^2+^ dynamics can respond rapidly to bioelectrical inputs and participate in mesenchymal cell differentiation, ECM regulation, and cellular stress responses [18,19]. Calcineurin is a Ca^2+^/calmodulin-dependent phosphatase, whereas NFAT proteins function as downstream Ca^2+^-responsive transcriptional regulators involved in cell-fate and matrix-remodeling programs [20]. We therefore examined whether microcurrent exposure was associated with altered intracellular Ca^2+^ responses and NFAT-related signaling. Because the upstream source of the Ca^2+^ response and the direct transcriptional targets of NFAT were not comprehensively resolved, the present mechanistic analyses were intended to assess pathway involvement rather than establish a complete linear causal mechanism.

Here, we established a microcurrent-assisted BMSC-derived CMT culture system and evaluated continuous unidirectional direct-current stimulation at 0, 50, 100, 150, 250, and 500 μA. This exploratory intensity-screening design was used to characterize stimulation-dependent changes in BMSC viability, chondrogenic differentiation, CMT morphology, cartilage-associated matrix formation, phenotype-related markers, and mechanical properties. The most favorable tested condition was identified from the overall convergence of molecular, biochemical, morphological, and mechanical outcomes rather than from a prospectively prespecified single optimization endpoint. Accordingly, the identified condition should be interpreted as the best-performing tested condition under the present experimental configuration, rather than as a universally optimal microcurrent dose. Ca^2+^ imaging, pharmacological interference, and NFATc1 localization analyses were further used to investigate the involvement of Ca^2+^/calcineurin/NFAT-related signaling.

Finally, CMTs preconditioned under selected microcurrent conditions were implanted subcutaneously into nude mice to determine whether the cartilage-like phenotype established *in vitro* persisted during short-term ectopic implantation. This model was intended as a proof-of-concept assessment of post-preconditioning tissue behavior, rather than as a model of orthotopic cartilage-defect repair. Overall, we examined microcurrent stimulation as an intensity-dependent bioelectrical cue for scaffold-free BMSC-derived CMT formation and emphasizes the need to balance favorable chondrogenic and matrix-associated responses against the adverse effects of excessive stimulation. These findings provide a basis for subsequent optimization and longer-term evaluation in clinically relevant orthotopic cartilage-defect models.

## 2 Materials and methods

### 2.1 BMSC isolation, culture, and characterization

All animal procedures were approved by the Institutional Animal Care and Use Committee of Shanghai Pulmonary Hospital and performed in accordance with institutional guidelines. Bone marrow was obtained from 15 individual male New Zealand white rabbits (4–6 weeks old, 0.8–1.2 kg; Shanghai Yunde Biotechnology Co., Ltd., Shanghai, China). Five independent cell-isolation/experimental runs collectively involved these 15 donors. Cells from different animals were maintained separately and were never pooled. Each donor-derived preparation was subsequently distributed across all six stimulation conditions, resulting in a matched within-donor design.

Bone marrow was flushed from the femurs and tibias with phosphate-buffered saline (PBS; Sigma, USA) containing 1% penicillin–streptomycin, filtered through a 70-μm strainer, and centrifuged at 300 × g for 5 min. Adherent cells were expanded in low-glucose dulbecco's modified eagle medium (DMEM; Sigma, USA) containing 10% fetal bovine serum and 1% penicillin–streptomycin at 37°C and 5% CO_2_, with medium replacement every 2–3 days.

Passage-3 (P3) cells were used throughout the study. Their BMSC phenotype was characterized by adherent spindle-shaped morphology and crystal-violet staining; flow cytometry for CD29, CD44, CD90, CD105, CD34, and CD45; and adipogenic, osteogenic, and chondrogenic differentiation assessed by Oil Red O, Alizarin Red, and Alcian Blue staining, respectively. All P3 preparations used experimentally were negative for mycoplasma contamination.

Chondrogenic induction medium consisted of high-glucose DMEM supplemented with 10 ng/mL transforming growth factor β3 (TGF-β3), 100 nM dexamethasone, 50 μg/mL ascorbic acid, 1 mM sodium pyruvate, 40 μg/mL proline, 1% insulin–transferrin–selenium, and 1% penicillin–streptomycin.

### 2.2 Microcurrent stimulation system and experimental design

Microcurrent stimulation was performed in sterile flat-bottom 12-well tissue-culture plates using a programmable constant-current source (Model 6221, Keithley Instruments, Cleveland, OH, USA) operated in continuous unidirectional direct-current mode. Each well constituted an electrically independent stimulation circuit and used a dedicated pair of rectangular platinum electrodes; wells were not connected in series or parallel (Fig. S1).

The immersed/exposed portion of each electrode measured 10 × 5 mm (50 mm^2^), the inter-electrode distance was approximately 15 mm, and each well contained 2 mL of culture medium. Cells or CMTs were maintained in the central culture region between the electrodes. The anode and cathode positions were fixed throughout stimulation and were not reversed, and electrode orientation and sample position were kept consistent across groups.

Samples were exposed to 0, 50, 100, 150, 250, or 500 μA. Based on the exposed area of one electrode, nominal electrode current density (*J* = *I*/*A*) was 0, 1, 2, 3, 5, and 10 μA/mm^2^, respectively, equivalent to 0, 100, 200, 300, 500, and 1000 μA/cm^2^. These values represent nominal electrode current densities, not spatially resolved local current densities experienced by individual cells or CMTs. The detailed electrical stimulation parameters used for BMSC-derived CMT culture are provided in Table S1.

At 150 μA, the measured potential difference across the stimulation system was approximately 0.3 V. Using the 15-mm inter-electrode distance, the estimated bulk-average electric field was approximately 20 V/m (0.2 V/cm), and the apparent system resistance calculated from the voltage/current ratio was approximately 2.0 kΩ. These are configuration-level descriptors and should not be interpreted as uniform local electrical fields or intrinsic medium resistance. Voltage was not systematically recorded for the other current conditions, and medium conductivity was not directly measured; therefore, unmeasured values were not retrospectively extrapolated.

The constant-current source maintained the prescribed nominal current by adjusting output voltage. Continuous voltage-drift/electrode-polarization measurements were not recorded. Unless otherwise stated, stimulation was applied 24 h/day throughout chondrogenic induction, except for brief interruptions during medium replacement; current was stopped during medium exchange and resumed immediately afterward. The 0 μA sham group contained the identical electrode configuration for the same duration and underwent the same handling but received no current output.

Each well used the same dedicated electrode pair throughout the experiment. Electrodes were sterilized before initial use and aseptically cleaned/re-sterilized during medium replacement before being returned to the same well; electrodes were not exchanged between treatment groups.

The six-intensity experiment was an exploratory intensity screen; no single primary optimization endpoint was prospectively prespecified. The 150 μA condition was subsequently selected as the best-performing tested condition based on convergence of molecular, biochemical, morphological, and mechanical outcomes under this defined electrical configuration.

### 2.3 Bulk culture conditions and reactive oxygen species (ROS) assessment

Bulk medium temperature and pH were measured in all six stimulation groups on days 1, 5, and 9. Cellular ROS were assessed on day 5 using a 2′,7′-dichlorodihydrofluorescein diacetate (DCFH-DA) assay (Beyotime Biotechnology, Shanghai, China), with fluorescence normalized to the 0 μA group.

These measurements evaluated gross thermal, acid–base, and oxidative conditions. Electrode-adjacent pH/redox gradients, medium conductivity and osmolality, prolonged electrode polarization, gas evolution or precipitation, platinum dissolution, and other localized or cumulative electrochemical products were not systematically quantified. A conditioned-medium control was not included. Accordingly, these measurements were not used to exclude localized electrochemical effects of continuous direct current (DC) stimulation.

### 2.4 DNA content and metabolic activity in 2D culture

DNA content was quantified on day 5 using a dsDNA assay (Beyotime Biotechnology). Metabolic activity was evaluated using Cell Counting Kit-8 (CCK-8; Beyotime Biotechnology) on days 0, 1, 3, 5, 9, and 11. After a 2-h incubation with CCK-8 reagent, absorbance was measured at 450 nm.

### 2.5 Viability and proliferation in 2D culture

Cell viability was evaluated by Calcein-AM/propidium iodide Live/Dead staining (Beyotime Biotechnology) on days 1, 3, 5, 7, and 9, with quantitative analysis performed on day 9.

DNA synthesis was assessed on day 9 by 5-ethynyl-2′-deoxyuridine (EdU) incorporation. Proliferative activity was additionally evaluated by Ki67 immunofluorescence. EdU- and Ki67-positive rates were calculated relative to total DAPI-positive nuclei. Images used for intergroup comparisons were acquired under identical settings.

### 2.6 Generation of BMSC-derived CMTs

BMSC-derived CMTs were operationally defined as scaffold-free 3D BMSC aggregates generated under chondrogenic induction that develop cartilage-like ECM, without implying the multicellular complexity or developmental organization of classical organoids.

P3 rabbit BMSCs were resuspended in chondrogenic medium and seeded into 1000-μm-diameter round-bottom microwells at 1 × 10^5^ cells per microwell. Following aggregation, CMTs were maintained at 37°C and 5% CO_2_ and exposed to 0, 50, 100, 150, 250, or 500 μA throughout induction as described above (Fig. S2).

Viability and early apoptosis were assessed on days 3 and 7, whereas the principal morphological, histological, biochemical, molecular, and mechanical endpoints were assessed on day 14. Thus, the present design evaluated the cumulative effect of sustained stimulation rather than discrete early-versus late-stage stimulation windows.

CMT diameter and circularity were quantified from light-microscopy images. Shrinkage was calculated as:

Shrinkage(%)=[1000−CMTdiameter(μm)]/1000×100%.

### 2.7 Viability, apoptosis, and proliferation-related assays in CMTs

CMT viability was assessed on days 3 and 7 using Live/Dead staining, and apoptosis was evaluated at the same time points using a Terminal deoxynucleotidyl transferase dUTP Nick-End Labeling (TUNEL) assay (Beyotime Biotechnology). TUNEL-positive area was quantified using ImageJ.

At day 14, apoptosis-related signaling was assessed by Western blotting for cleaved caspase-3, Bax, and Bcl-2, including calculation of the Bax/Bcl-2 ratio. Proliferation-related responses were evaluated by PCNA and Cyclin D1 Western blotting and by reverse transcription quantitative polymerase chain reaction (RT-qPCR) of *Ccnd1*, *Mki67*, and *Pcna* at the indicated time points.

### 2.8 Histological and immunohistochemical staining

Day-14 CMTs were fixed in 4% paraformaldehyde, paraffin-embedded, and sectioned at 6 μm. Hematoxylin and eosin (H&E), toluidine blue, and safranin O staining were performed to assess morphology and proteoglycan-rich matrix.

For COL II immunohistochemistry, sections were deparaffinized, subjected to antigen retrieval and blocking of endogenous peroxidase activity, blocked with 5% Bovine Serum Albumin (BSA; Sigma, USA), and incubated overnight at 4°C with anti-COL II antibody (Abcam, ab34712; 1:200). Immunoreactivity was visualized using HRP/DAB with hematoxylin counterstaining. COL II staining was performed under standardized conditions, and images used for intergroup comparison were acquired using consistent imaging settings. The same procedures were applied to post-implantation explants.

### 2.9 Reverse transcription-quantitative polymerase chain reaction (RT-qPCR)

Total RNA was isolated using TRIzol reagent (Invitrogen), and cDNA was synthesized using the PrimeScript RT Reagent Kit with gDNA Eraser (Takara Bio). RT-qPCR was performed using TB Green Premix Ex *Taq*II on a QuantStudio 5 system.

In 2D BMSCs, *Col2a1*, *Sox9*, and *Acan* were analyzed on day 12. In CMTs, Ccnd1, Mki67, and Pcna were evaluated on days 3, 7, and 14. Day-14 CMTs were additionally analyzed across all six current conditions for chondrogenic (*Col2a1*, *Sox9*, and *Acan*), fibrotic/hypertrophic (*Col1a1*, *Col10a1*, *Runx2*), and catabolic (*Mmp13*, *Adamts5*) genes.

Gapdh was used as the reference gene. Raw Gapdh Cq values were re-examined across stimulation conditions and showed no systematic current-dependent shift. Relative expression was calculated using the 2^−ΔΔCt^ method. Melt-curve analysis showed a single dominant peak for each primer pair without obvious secondary peaks. Serial-dilution standard curves were not generated; therefore, primer-specific amplification efficiencies were not experimentally determined. Rabbit (*Oryctolagus cuniculus*) primer sequences are provided in Supplementary Table S2.

### 2.10 Immunofluorescence and pharmacological interference

Immunofluorescence was used to evaluate COL II, COMP, and ACAN in 2D BMSCs on day 12 and in CMTs on day 14. Day-14 CMTs were additionally examined for COL I, COL X, RUNX2, MMP13, ADAMTS5, iNOS, and COX-2. COL X represents the protein product encoded by *Col10a1*. Fluorescence-positive areas were reported as immunoreactivity, rather than as direct measurements of protein production.

Samples were fixed with 4% paraformaldehyde, permeabilized with 0.3% Triton X-100, blocked with 5% BSA, and incubated with the appropriate primary antibody overnight at 4°C. Samples were then incubated with Alexa Fluor-conjugated secondary antibodies and counterstained with 4′,6-Diamidino-2-Phenylindole (DAPI; Sigma, USA). Detailed antibody information, including catalog number, dilution, host species, manufacturer-reported species reactivity, expected molecular weight, and basis for application to rabbit samples, is provided in Table S3. Manufacturer-tested, manufacturer-cited/listed, and sequence-homology-predicted rabbit reactivity were distinguished.

For NFATc1 analysis, CMTs were assigned to 0 μA, 150 μA, 150 μA + verapamil, 150 μA + BAPTA-AM, 150 μA + cyclosporin A (CsA), or 500 μA. Verapamil (10 μM), BAPTA-AM (10 μM), or CsA (1 μM) was added 1 h before stimulation and maintained thereafter. The final dimethyl sulfoxide (DMSO; Sigma, USA) concentration was 0.1% (v/v) in all inhibitor-containing groups. A matched DMSO vehicle-control group and unstimulated inhibitor-only groups were not included in the original experimental design.

The NFATc1 nuclear/cytoplasmic fluorescence ratio and the percentage of cells showing predominant nuclear localization were quantified. These measurements were interpreted as indicators of NFATc1 nuclear translocation, not direct measurements of NFAT transcriptional activity.

### 2.11 Western blot analysis

Samples were lysed in RIPA buffer containing protease and phosphatase inhibitors. Protein concentration was determined by BCA assay, and equal amounts of protein were separated by SDS-PAGE, transferred to PVDF membranes, and analyzed by chemiluminescence.

Proteins examined were COL II (calculated 134 kDa; apparent band ∼200 kDa), SOX9 (∼56 kDa), ACAN (∼250 kDa), cleaved caspase-3 (17/19 kDa), PCNA (calculated ∼29 kDa; apparent/modified form ∼36 kDa), Cyclin D1 (∼34 kDa), NFATc1 (calculated isoforms ∼78 and 101 kDa; apparent range ∼82–140 kDa), Bax (∼21 kDa), Bcl-2 (∼26 kDa), and β-actin (∼42 kDa). Antibody details are provided in Table S3.

COL II, SOX9, and ACAN were analyzed in 2D BMSCs on day 12. Cleaved caspase-3, PCNA, Cyclin D1, total NFATc1, Bax, and Bcl-2 were examined in day-14 CMTs. NFATc1 Western blotting used whole-cell lysates and therefore reflected total NFATc1 abundance rather than activation or nuclear translocation.

Band intensities were quantified from original chemiluminescent images using ImageJ after background subtraction and normalized to the corresponding β-actin signal from the same biological sample. Images with obvious saturation were excluded. Formal serial-loading validation of the linear detection range was not performed. Detailed information regarding antibody host species, manufacturer-reported species reactivity, expected molecular weight, and the basis for application to rabbit-derived samples is provided in Table S3.

### 2.12 Biochemical quantification of COL II and GAG

Sulfated GAG was quantified using the Blyscan Dimethylmethylene Blue assay (Biocolor, Carrickfergus, UK). Native type II collagen was measured using the Type II Collagen Detection ELISA Kit, Multi-Species (Cat. No. 6018; Chondrex, Inc., Woodinville, WA, USA), which preferentially detects native, undenatured COL II and was not used to quantify specific COL II degradation products.

For *in vitro* CMTs, native COL II content and total sulfated GAG content were quantified. DNA content was measured in parallel, and COL II/DNA and GAG/DNA ratios were additionally calculated. For post-implantation samples, each complete explant from one mouse constituted one biological sample; COL II and GAG were reported as total content per explant without normalization to wet weight, dry weight, or DNA. The assay did not establish donor-versus-host origin of matrix in the xenogeneic implantation model.

### 2.13 ATP-based metabolic activity assay

CMT metabolic activity was assessed on days 3, 7, and 14 using the CellTiter-Glo 3D assay (Promega, Madison, WI, USA). Samples were lysed according to the manufacturer's instructions, and luminescence was measured using a SpectraMax iD3 plate reader. Values were normalized to the corresponding 0 μA group at each time point.

### 2.14 Mechanical testing and hydration analysis

Day-14 CMTs were tested using an Instron 5943 equipped with a 5 N load cell and 10-mm parallel compression platens. Samples were equilibrated in PBS for 15 min at 22–25°C. Two perpendicular transverse diameters (*d*_*1*_,*d*_*2*_) and initial height (*h*_*0*_) were measured for each hydrated specimen. Because CMTs were not assumed to be perfect cylinders, initial cross-sectional area was approximated individually as:

$$A_{0}=\left(\pi d_{1}d_{2}\right)/4\text{.}$$

For unconfined compression, a 5 mN preload was applied before compression at 0.5 mm/min to 20% engineering strain. Engineering stress and strain were calculated as:

$$\sigma =F/A_{0},\varepsilon =\Delta h/h_{0}\text{.}$$

The apparent compressive modulus was obtained by least-squares linear regression over the predefined 5–15% strain interval, with *R*^*2*^ calculated for each fit.

For dynamic testing, specimens were maintained at a 10% static compressive offset and subjected to ±1% sinusoidal strain at 1 Hz for 10 cycles. No separate cyclic preconditioning was performed. The final five stable cycles were analyzed, and storage modulus was calculated as:

$$E^{\prime }=\left(\sigma_{0}/\varepsilon_{0}\right)\cos\;\delta \text{.}$$

The same specimen-specific geometric approach was used for post-implantation tissue. Calculated moduli were therefore interpreted as apparent geometry-dependent bulk properties.

For hydration measurements, samples were weighed in the hydrated state and after freeze-drying to constant mass. Water content and wet/dry ratio were calculated from wet and dry weights.

### 2.15 Intracellular Ca^2+^ imaging

Ca^2+^ imaging was performed on dissociated cells derived from CMTs, rather than intact 3D CMTs. Cells from the 0, 150, and 500 μA groups were loaded with Fluo-4 AM for 30 min at 37°C, washed with HBSS, and transferred to phenol red-free medium.

Time-lapse imaging was performed using a ZEISS Axio Observer 7 inverted fluorescence microscope with an Axiocam 705 mono camera and Plan-Apochromat 20×/0.8 NA objective. Fluo-4 fluorescence was acquired using 470/40-nm excitation and 525/50-nm emission filters at 5 frames/s with a 50-ms exposure time, using identical acquisition settings among groups.

A 5-s baseline preceded a 60-s recording after stimulation onset. Minor field drift was corrected by image registration, and the same cells were tracked throughout each recording. *F*_*0*_ was defined as the mean baseline fluorescence. Cells were classified as responsive when *ΔF/F*_*0*_ exceeded the baseline mean by > 3 SD for at least two consecutive frames. Responsive-cell ratio and 0–60-s AUC were calculated.

For each donor and condition, two independently generated CMT-derived samples were analyzed, with three non-overlapping fields per sample and approximately 20–30 cells per field. Cellular data were hierarchically averaged to obtain one donor-level value; cells, fields, and CMT-derived samples were treated as subsamples, and the rabbit donor was the biological replicate (n = 15). Analysis was performed blinded to treatment allocation. The 0, 150, and 500 μA groups represented baseline, the selected favorable condition, and excessive stimulation, respectively; the experiment was therefore a focused mechanistic comparison rather than a complete Ca^2+^ dose-response analysis.

### 2.16 Xenogeneic subcutaneous implantation

The short-term *in vivo* behavior of microcurrent-preconditioned CMTs was evaluated using a xenogeneic ectopic subcutaneous implantation model. Rabbit BMSC-derived CMTs from the 0, 150, and 500 μA groups were collected after 14 days, washed with sterile PBS, and prepared at 50 CMTs per 100 μL.

Fifteen four-week-old male BALB/c nude mice (18–22 g; Shanghai Yunde Biotechnology Co., Ltd.) were allocated to three groups (n = 5 mice/group). Each mouse received one treatment condition at one standardized dorsal subcutaneous site; no mouse received multiple conditions or implantation sites. Thus, the individual mouse was the independent experimental unit. Implantation and harvesting were not formally blinded; however, subsequent quantitative image analysis was performed on coded samples by an assessor blinded to treatment allocation.

General anesthesia was induced with 3% isoflurane and maintained with 1.5–2.0% isoflurane in oxygen. Adequate anesthesia was confirmed by absence of the pedal-withdrawal reflex. Buprenorphine (0.05 mg/kg, subcutaneously) was administered approximately 30 min before implantation and every 12 h for 24 h thereafter. Animals recovered with thermal support and were returned to their cages after regaining spontaneous movement.

The dorsal skin was disinfected, and 100 μL of suspension containing 50 CMTs was implanted subcutaneously. Animals were monitored at least daily for 2 weeks, with additional observations during the first 24–48 h, including activity, posture, grooming, food/water intake, body condition, wound healing, local swelling/infection, and signs of pain or distress. Humane endpoints included >20% body-weight loss, persistent severe hypoactivity or inability to access food/water, progressive wound complications, marked infection/necrosis, or persistent uncontrolled pain/distress.

After 2 weeks, explants were harvested for gross examination, histology, COL II immunohistochemistry, biochemical COL II/GAG measurements, and apparent compressive-modulus testing. All 15 mice completed the experiment, with no implant loss or exclusion.

Because rabbit-derived CMTs were implanted into immunodeficient mice, donor cells were not lineage-traced or distinguished from host cells by species-specific analysis. Post-implantation hypertrophic, osteogenic/mineralization, angiogenic, host inflammatory-response, and vascular-invasion markers were not systematically evaluated. Accordingly, this experiment was designed as a short-term xenogeneic ectopic proof-of-concept assessment, not as an orthotopic cartilage-defect repair model.

### 2.17 Image quantification and statistical analysis

All image-based analyses were performed using ImageJ. For *in vitro* section-based analyses, three CMTs per donor-condition and three nonadjacent sections per CMT were evaluated. Sections encompassing the central/maximal cross-sectional region were selected using the same predefined rule across groups. Where possible, the entire CMT cross-section was analyzed; otherwise, fields were obtained using a predefined systematic sampling pattern rather than staining appearance. For 2D immunofluorescence, three non-overlapping fields from two technical wells were analyzed per donor-condition. For *in vivo* samples, three nonadjacent sections from each explant were evaluated.

The same background correction, region-of-interest definition, thresholding criteria, and acquisition settings were applied within each marker. Samples from different treatment groups were processed in parallel within staining batches whenever possible. Image files were coded before quantitative analysis, and the investigator performing image quantification was blinded to treatment identity.

For *in vitro* experiments, the individual rabbit donor was the biological experimental unit (n = 15). Five experimental runs collectively involved the 15 donors, but run was not treated as the inferential unit. Cells from different donors were not pooled, and each donor was represented across all six stimulation conditions. Multiple wells, CMTs, sections, fields, cells, or repeated measurements from the same donor-condition were treated as technical subsamples and averaged to generate one donor-level value.

Single-time-point *in vitro* endpoints were analyzed using linear mixed-effects models, with stimulation condition as a fixed effect and donor as a random intercept. For longitudinal endpoints, stimulation condition, time, and their interaction were included as fixed effects, with donor as a random effect. Pairwise contrasts of estimated marginal means were multiplicity-adjusted.

Model assumptions were evaluated using Q–Q plots and the Shapiro–Wilk test for residual normality and using residual-versus-fitted plots and, where applicable, the Brown–Forsythe test for homogeneity of variance. Appropriate transformation was considered when required. Matched datasets remaining substantially non-normal were analyzed using the Friedman test with multiplicity-adjusted paired comparisons.

For the *in vivo* experiment, the mouse was the independent unit (n = 5/group). Parametric comparisons among the 0, 150, and 500 μA groups were performed using one-way ANOVA followed by Tukey's multiple-comparisons test; the Kruskal–Wallis test with Dunn's correction was used when parametric assumptions were not satisfied.

For principal intensity-response endpoints, planned contrasts included 150 μA versus 0, 100, and 250 μA. Other pairwise comparisons were treated as secondary/exploratory and adjusted for multiplicity. False-discovery-rate control using the Benjamini–Hochberg procedure was applied to predefined families of related endpoints where appropriate.

Exact multiplicity-adjusted P values are reported for key comparisons, particularly 150 μA versus 0 μA, together with estimated effect differences and 95% confidence intervals where appropriate. Figure legends specify the statistical test and number/identity of independent biological replicates. Data are presented as mean ± SD unless otherwise stated, and an adjusted two-sided *P* value < 0.05 was considered statistically significant. Statistical annotations were defined as follows: ns, not significant; *P < 0.05; **P < 0.01; ***P < 0.001; and ****P < 0.0001.

## 3 Results and discussion

### 3.1 Microcurrent stimulation maintained BMSC viability while modulating proliferative activity during 2D chondrogenic induction

Before evaluating cartilage-specific differentiation, we first determined whether direct microcurrent stimulation affected the basic cellular status of BMSCs under 2D chondrogenic induction conditions (Fig. 1A). BMSCs were exposed to continuous direct microcurrent stimulation at 0, 50, 100, 150, 250, and 500 μA, and cell viability, DNA content, metabolic activity, and proliferation-related markers were examined. Relative DNA content and CCK-8 analysis showed that microcurrent stimulation did not induce a simple linear increase in cell number or metabolic activity on day 5 (Fig. 1B and C). Consistently, Live/Dead staining from days 1 to 9 demonstrated that BMSCs remained largely viable across the stimulation groups, indicating that the tested current range did not cause overt acute cytotoxicity in 2D culture (Fig. 1D). Quantitative analysis of cell viability on day 9 further confirmed that most cells remained viable after microcurrent stimulation, although viability varied among different current-intensity groups (Fig. 1E). Supplementary CCK-8 analysis over an extended time course from day 0 to day 11 further supported that microcurrent stimulation did not primarily act by nonselectively accelerating cell expansion (Fig. S3).

**Fig. 1:**
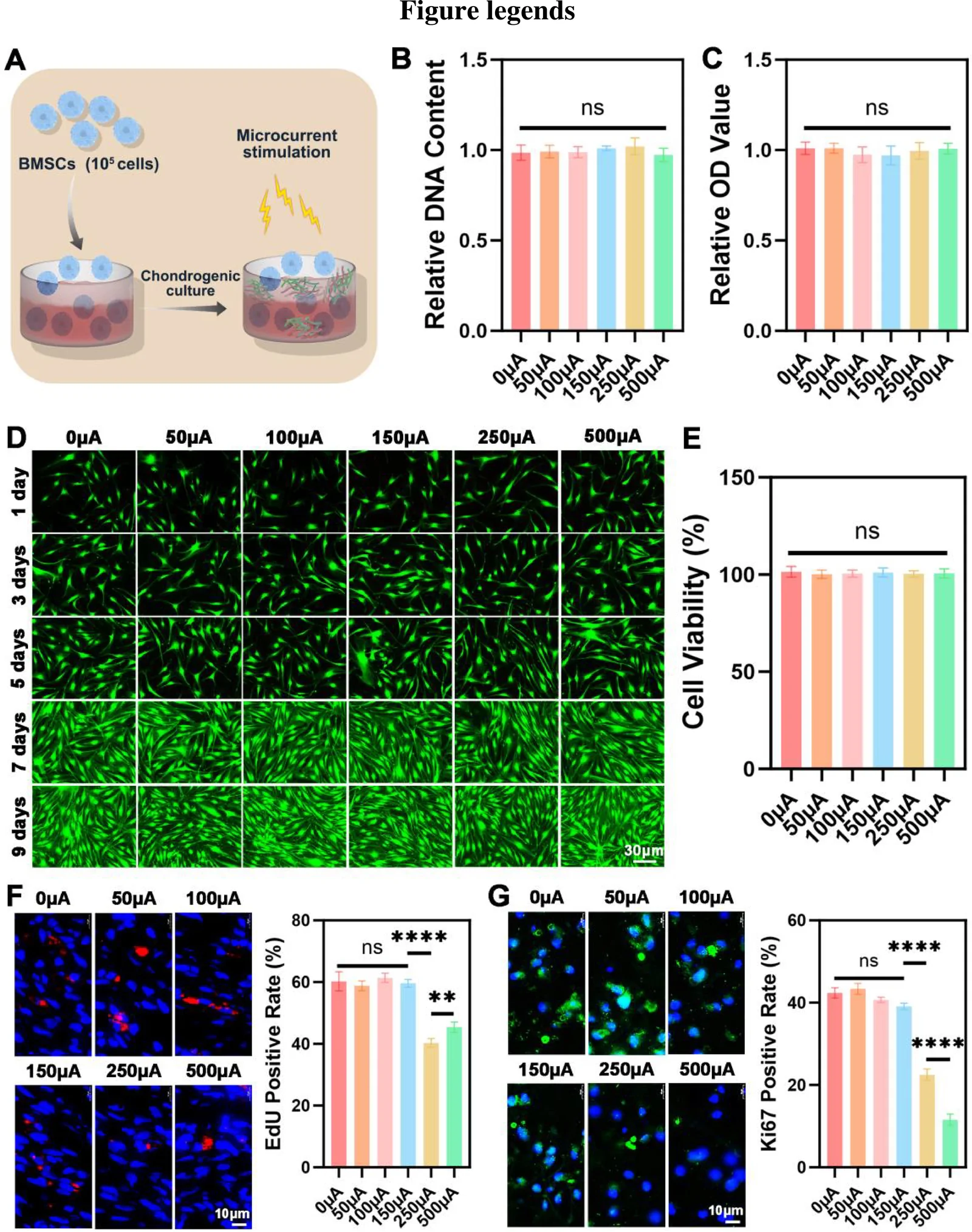
**Microcurrent stimulation did not markedly affect the viability of 2D-cultured BMSCs but modulated their proliferative activity in an intensity-dependent manner. (A)** Schematic illustration of the experimental design for chondrogenic induction of BMSCs in a 2D culture system under microcurrent stimulation. **(B)** Relative DNA content and **(C)** optical density (OD) values determined by the CCK-8 assay in 2D-cultured BMSCs exposed to 0, 50, 100, 150, 250, and 500 μA microcurrent stimulation at day 5. **(D)** Live/Dead staining of 2D-cultured BMSCs in the 0, 50, 100, 150, 250, and 500 μA groups at days 1, 3, 5, 7, and 9. **(E)** Quantification of cell viability in 2D-cultured BMSCs at day 9. **(F)** EdU immunofluorescence staining and quantitative analysis of the EdU-positive rate in 2D-cultured BMSCs at day 9. **(G)** Ki67 immunofluorescence staining and quantitative analysis of the Ki67-positive rate in 2D-cultured BMSCs at day 9. Data are presented as mean ± SD (n = 15 independent rabbit donors). Five independent experimental runs were performed, collectively involving the 15 donors. For each donor and stimulation condition, measurements from technical culture wells and microscopic fields were averaged to generate a single donor-level value before statistical analysis. Individual wells, images, fields, and cells were not treated as independent biological replicates. Stimulation conditions were matched within donors. ns, not significant; **P < 0.01; ****P < 0.0001.

Although overall viability was preserved, we further examined DNA synthesis and proliferation-associated nuclear marker expression to clarify whether microcurrent stimulation affected the proliferative status of BMSCs. EdU incorporation was used to identify cells undergoing active DNA synthesis during S phase [21]. Representative EdU staining images showed comparable EdU-positive signals in the 0, 50, 100, and 150 μA groups on day 9, whereas the EdU-positive population was markedly reduced in the 250 and 500 μA groups. Quantitative analysis of the EdU-positive rate confirmed that low-to-moderate microcurrent stimulation (≤150 μA) did not significantly affect DNA synthesis activity, while high-intensity stimulation (≥250 μA) significantly suppressed EdU incorporation (Fig. 1F). Ki67 staining was then performed as an independent proliferation-related marker, because Ki67 is expressed in actively cycling cells but absent in quiescent cells [22]. Consistent with the EdU results, representative Ki67 immunofluorescence images and quantification of the Ki67-positive rate showed no obvious difference among the 0, 50, 100, and 150 μA groups, but a pronounced decrease in the 250 and 500 μA groups on day 9 (Fig. 1G). These findings indicate that microcurrent stimulation within the low-to-moderate range preserved BMSC proliferative activity, whereas excessive stimulation impaired proliferation.

This distinction is important because the subsequent enhancement of cartilage matrix formation in the 150 μA stimulation group should not be interpreted as a consequence of increased cell number or excessive proliferation. Instead, the data suggest that 150 μA stimulation maintained BMSC viability and proliferative capacity while creating a defined electrical stimulation environment associated with favorable chondrogenic regulation. In contrast, high-intensity stimulation at 250 and 500 μA suppressed proliferation, suggesting that excessive current may impose cellular stress or disturb homeostatic activity even in the absence of widespread acute cell death.

To assess potential bulk microenvironmental changes during stimulation, medium temperature, pH, and cellular ROS signals were examined during 2D induction. Temperature and pH measurements on days 1, 5, and 9 showed that microcurrent stimulation did not substantially alter the thermal or acid-base status of the culture medium across the tested current range (Fig. S4A and B). ROS levels were further assessed on day 5 during 2D chondrogenic induction, and no obvious increase in cellular ROS signal was observed across the tested stimulation conditions (Fig. S4C). Together with the temperature and pH measurements, these results indicate that microcurrent stimulation did not produce detectable alterations in bulk medium temperature, bulk pH, or the measured cellular ROS signal under the tested conditions. However, these measurements were not designed to resolve localized or cumulative electrochemical changes at the electrode–medium interface and therefore cannot exclude local pH or redox gradients, electrode polarization, changes in dissolved gases or ionic composition, or other electrode-associated electrochemical effects. Accordingly, the observed biological responses should be interpreted as arising from the overall electrical stimulation environment rather than as evidence of an exclusively bioelectrical mechanism.

### 3.2 Microcurrent stimulation modulated chondrogenic differentiation of 2D-cultured BMSCs in an intensity-dependent manner

After confirming cytocompatibility, we next investigated whether microcurrent stimulation promoted chondrogenic differentiation of BMSCs. RT-qPCR analysis showed that the chondrogenic genes *Col2a1*, *Sox9*, and *Acan* were regulated by stimulation intensity on day 12 (Fig. 2A). The expression of these markers increased under low-to-moderate microcurrent stimulation, with the most favorable response observed at 150 μA, whereas higher intensities (≥250 μA) did not further enhance chondrogenic gene expression. These results indicate that microcurrent stimulation did not exhibit a simple ‘more is better’ response; instead, it showed a non-monotonic intensity-dependent pattern, with the most favorable profile observed at 150 μA among the tested conditions.

**Fig. 2:**
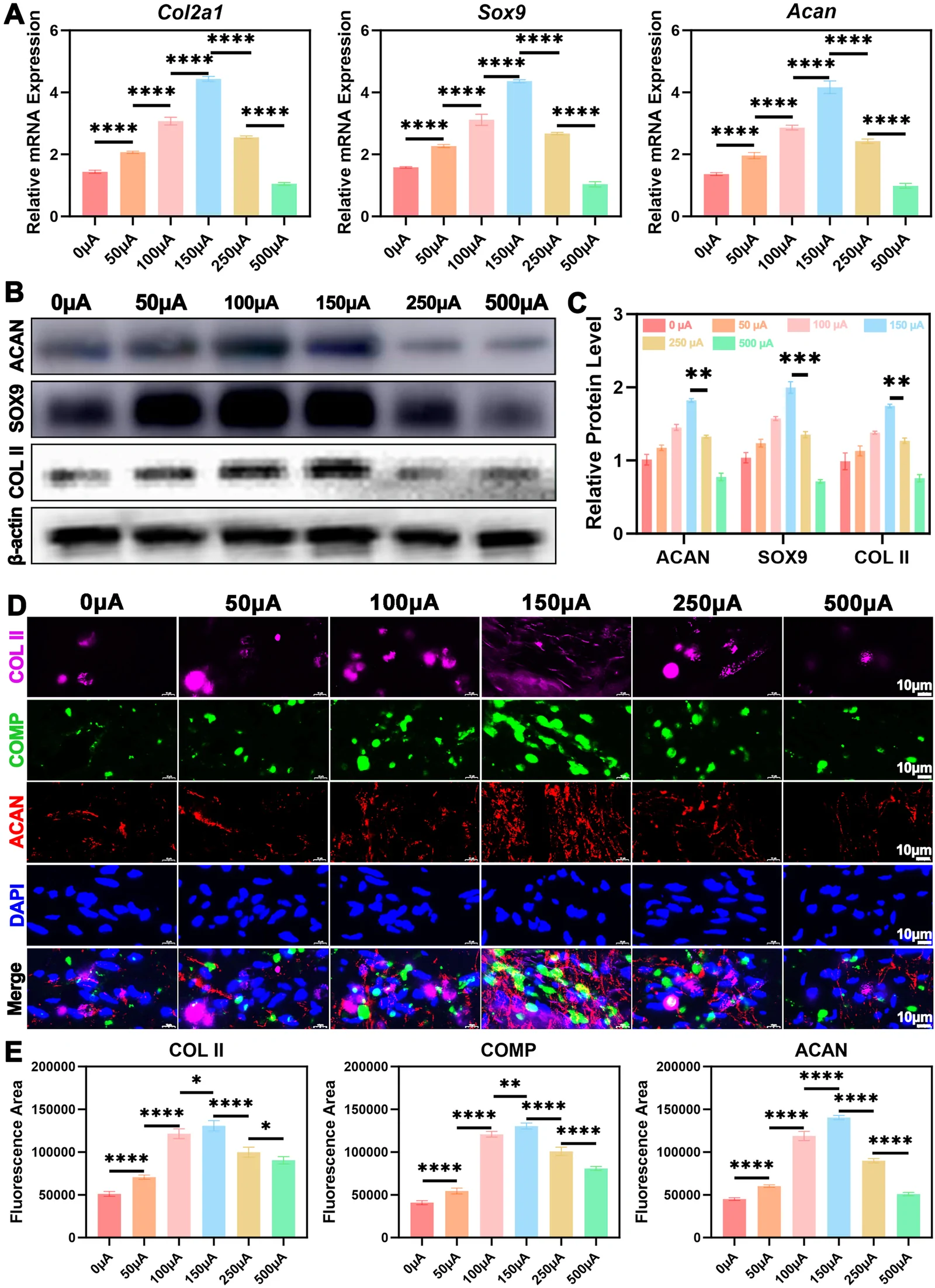
**Microcurrent stimulation enhanced the chondrogenic differentiation capacity of 2D-cultured BMSCs in an intensity-dependent manner. (A)** Relative mRNA expression levels of chondrogenic markers, including *Col2a1*, *Sox9*, and *Acan*, in 2D-cultured BMSCs exposed to 0, 50, 100, 150, 250, and 500 μA microcurrent stimulation at day 12, as determined by RT-qPCR. **(B)** Representative Western blot images showing the protein expression levels of COL II, SOX9, and ACAN in 2D-cultured BMSCs from each group at day 12, with β-actin used as the loading control. **(C)** Quantitative analysis of the relative protein expression levels of COL II, SOX9, and ACAN based on the Western blot results shown in **(B)**. **(D)** Representative immunofluorescence staining images of COL II, COMP, and ACAN in 2D-cultured BMSCs from each group at day 12. **(E)** Quantitative analysis of the fluorescence-positive areas of COL II, COMP, and ACAN based on the immunofluorescence staining shown in **(D)**. Data are presented as mean ± SD (n = 15 independent rabbit donors; stimulation conditions matched within donors). Five independent experimental runs collectively involved the 15 donors. Technical RT-qPCR reactions, Western blot measurements, and immunofluorescence fields obtained from the same donor-condition were averaged to generate one donor-level value. *P < 0.05; **P < 0.01; ***P < 0.001; ****P < 0.0001.

Western blotting further confirmed the transcriptional findings. Protein levels of COL II, SOX9, and ACAN were enhanced in response to appropriate microcurrent stimulation, with the most favorable chondrogenic marker profile observed at 150 μA among the tested conditions (Fig. 2B and C). Immunofluorescence staining of COL II, COMP, and ACAN showed a consistent matrix-level response, with stronger cartilage-related ECM signals observed in the low-to-moderate stimulation groups and the most prominent staining in the 150 μA group (Fig. 2D). Quantitative analysis of the fluorescence-positive areas further confirmed that COL II, COMP, and ACAN deposition was significantly increased by 150 μA stimulation, whereas excessive stimulation reduced these chondrogenic ECM signals (Fig. 2E). These proteins represent key components of hyaline cartilage-like ECM, and their coordinated upregulation suggests that microcurrent stimulation promotes a cartilage-forming phenotype rather than selectively increasing a single marker [23].

Among the tested intensities, 150 μA produced the most consistently favorable overall chondrogenic response. Because no single optimization endpoint was prospectively prespecified, 150 μA was selected, on an exploratory basis, as the best-performing tested condition for subsequent mechanistic analyses based on convergent molecular and matrix-related findings rather than on a predefined dose-selection criterion. Importantly, the favorable chondrogenic response at 150 μA was not accompanied by obvious cytotoxicity or enhanced proliferative activity, suggesting that the subsequent improvement in cartilage-related outcomes was not simply attributable to increased cell number.

These 2D findings provided the basis for subsequent 3D microtissue experiments. Because cartilage formation is inherently a 3D process involving cell condensation, cell–cell interactions, and extracellular matrix retention [24], we next examined whether the favorable chondrogenic response observed at 150 μA could be reproduced during the formation of BMSC-derived CMTs. However, because microcurrent stimulation was applied continuously throughout the induction period, the present data do not distinguish whether its effects arose predominantly during early lineage commitment or accumulated across later differentiation and matrix-formation stages.

### 3.3 The 150 μA condition was associated with favorable CMT morphology, cartilage-associated matrix deposition, and apparent mechanical properties

BMSC-derived **CMT**s were generated in microtissue microwells using 1 × 10^5^ cells per microwell and cultured under 3D chondrogenic conditions with microcurrent stimulation (Fig. 3A). On day 14, light microscopy revealed clear intensity-dependent differences in **CMT** morphology among the 0, 50, 100, 150, 250, and 500 μA groups (Fig. 3B). Quantitative analysis of CMT circularity and diameter showed a progressive improvement in morphology from 0 to 150 μA, with increased geometric regularity and a more compact construct size. In contrast, higher stimulation intensities, particularly 250 and 500 μA, impaired CMT formation and reduced morphological quality (Fig. 3C). Shrinkage analysis further indicated that microcurrent intensity influenced tissue contraction during microtissue maturation (Fig. S5). Since excessive shrinkage often reflects abnormal matrix remodeling, insufficient matrix retention, or altered cell-matrix tension, these morphological data provide an early indication that high-intensity stimulation may be unfavorable for stable cartilage-like tissue formation [25].

**Fig. 3:**
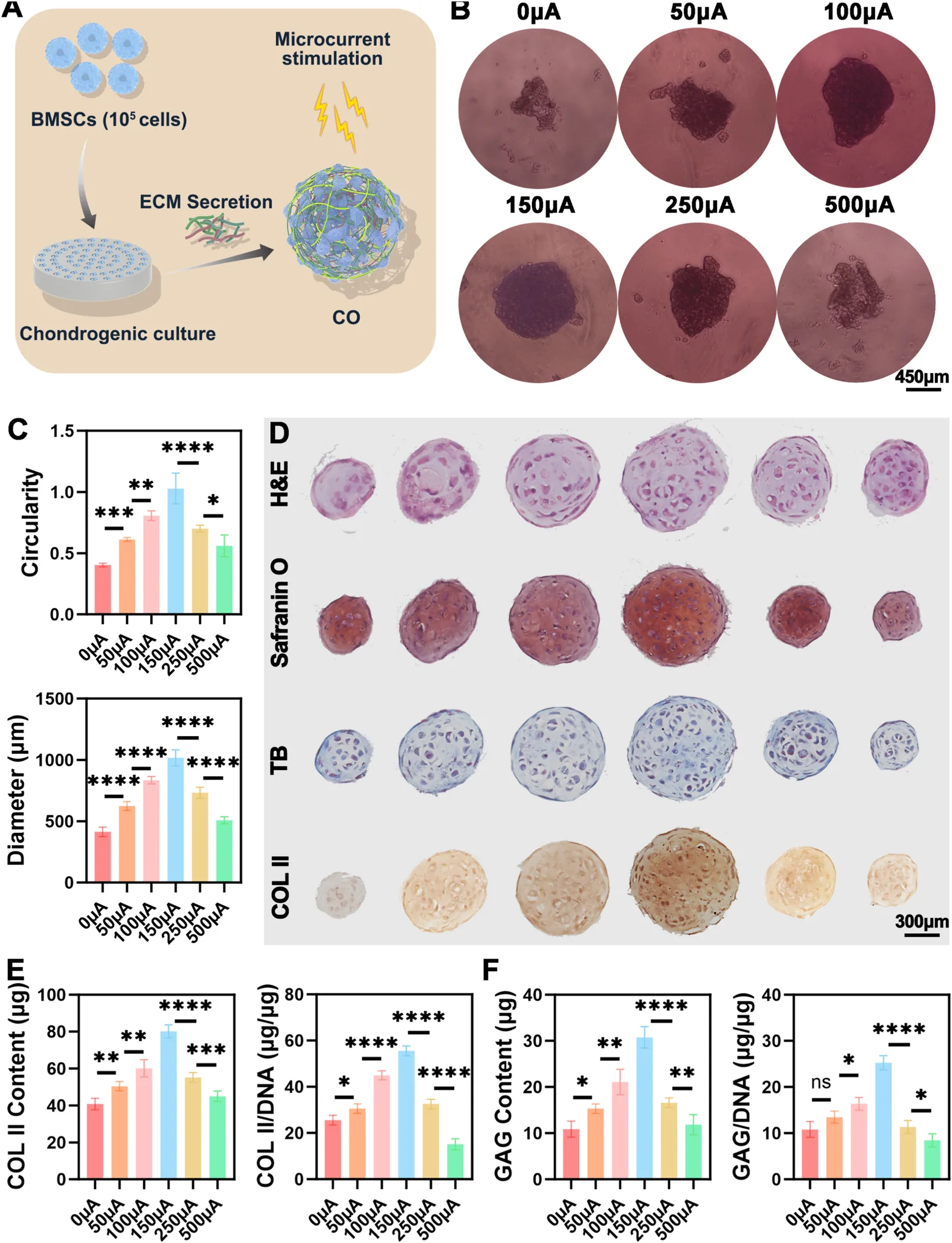
**Effects of different microcurrent stimulation intensities on cartilage-specific matrix formation in BMSC-derived CMTs on day 14. (A)** Schematic illustration of the experimental design. Briefly, 1 × 10^5^ BMSCs were seeded into each microtissue microwell and subsequently exposed to continuous direct microcurrent stimulation to promote CMT formation in a 3D chondrogenic culture system. **(B)** Representative light microscopy images of BMSC-derived CMTs in the 0, 50, 100, 150, 250, and 500 μA groups. **(C)** Quantitative analysis of the circularity and diameter of BMSC-derived CMTs in each group. **(D)** Representative H&E, Toluidine Blue (TB), Safranin O, and immunohistochemical COL II staining images of BMSC-derived CMTs from each group. **(E)** Quantitative analysis of COL II content and COL II/DNA level in BMSC-derived cartilage microtissues from each group. **(F)** Quantitative analysis of glycosaminoglycan (GAG) content and GAG/DNA level in BMSC-derived cartilage microtissues from each group. Data are presented as mean ± SD (n = 15 independent rabbit donors; stimulation conditions matched within donors). Multiple CMTs generated from the same donor under the same stimulation condition were treated as technical subsamples and were averaged to generate a single donor-level value before statistical analysis. *P < 0.05; **P < 0.01; ***P < 0.001; ****P < 0.0001**.** (For interpretation of the references to color in this figure legend, the reader is referred to the Web version of this article.)

To further evaluate whether these morphological and matrix-level changes translated into functional tissue properties, the mechanical and hydration-related characteristics of BMSC-derived CMTs were assessed on day 14. The compressive modulus and dynamic storage modulus increased progressively from 0 to 150 μA and declined when the stimulation intensity was further increased to 250 and 500 μA (Fig. S6A and B). These results indicate that 150 μA stimulation enhanced the mechanical competence of BMSC-derived CMTs, consistent with the increased COL II- and GAG-rich matrix deposition. Water content and wet-to-dry weight ratio were also affected by microcurrent intensity (Fig. S6C and D), suggesting that optimized stimulation improved hydration-related matrix organization, whereas excessive stimulation compromised tissue-level maturation.

Histological staining provided stronger evidence for cartilage-specific matrix formation (Fig. 3D). H&E staining showed progressively improved tissue organization from 0 to 150 μA, with the 150 μA group displaying a more compact and homogeneous cartilage-like structure. TB and Safranin O staining revealed a parallel increase in proteoglycan-rich matrix deposition across the 0–150 μA range. Immunohistochemical staining for COL II was also most prominent under optimized 150 μA microcurrent stimulation, whereas COL II staining began to decline when the current intensity was increased beyond 150 μA. Enlarged histological images further supported the presence of more compact and cartilage-like matrix organization in the favorable stimulation range (Fig. S7).

Biochemical quantification confirmed these histological observations. COL II content, COL II/DNA, GAG content, and GAG/DNA increased progressively from 0 to 150 μA and decreased in the higher-intensity groups, particularly at 250 and 500 μA (Fig. 3E and F). Normalization to DNA is particularly important because it shows that the improvement in matrix accumulation was not simply due to differences in cell number [26,27]. Instead, 150 μA stimulation enhanced the matrix-producing capacity of the cells within each CMT. Together, these results demonstrate that 150 μA microcurrent stimulation promoted both the morphological maturation and cartilage-specific ECM deposition of BMSC-derived CMTs.

By day 14, the favorable 150 μA condition was associated with greater cartilage-like matrix accumulation, improved CMT morphology, and enhanced apparent mechanical properties. These tissue-level outcomes are consistent with improved cartilage-like matrix formation. However, because the principal matrix-related endpoints were assessed at day 14 and microcurrent stimulation was applied continuously throughout induction, the present data do not establish when the chondrogenic advantage of the 150 μA condition first emerged. Although early cellular status was evaluated on days 3 and 7, these measurements were not designed to distinguish effects on early lineage commitment from those on subsequent extracellular-matrix accumulation.

### 3.4 Microcurrent stimulation regulated the molecular and matrix phenotype of BMSC-derived CMTs in an intensity-dependent manner

To further define the quality of the newly formed cartilage-like matrix, we examined chondrogenic ECM markers and proteins associated with dedifferentiation, hypertrophy, and matrix degradation. Immunofluorescence staining showed that COL II-, COMP-, and ACAN-positive signals/areas increased from 0 to 150 μA, with the strongest immunoreactivity observed under the 150 μA condition, whereas these cartilage-associated fluorescence signals decreased at 250 and 500 μA (Fig. 4A and B). The simultaneous elevation of these three matrix components suggests a coordinated chondrogenic response. COL II and ACAN are central structural and functional components of cartilage ECM [28], whereas COMP contributes to collagen fibril organization and matrix assembly [29]. Therefore, the concurrent increase in COL II, COMP, and ACAN immunoreactivity was consistent with a more cartilage-associated matrix profile.

**Fig. 4:**
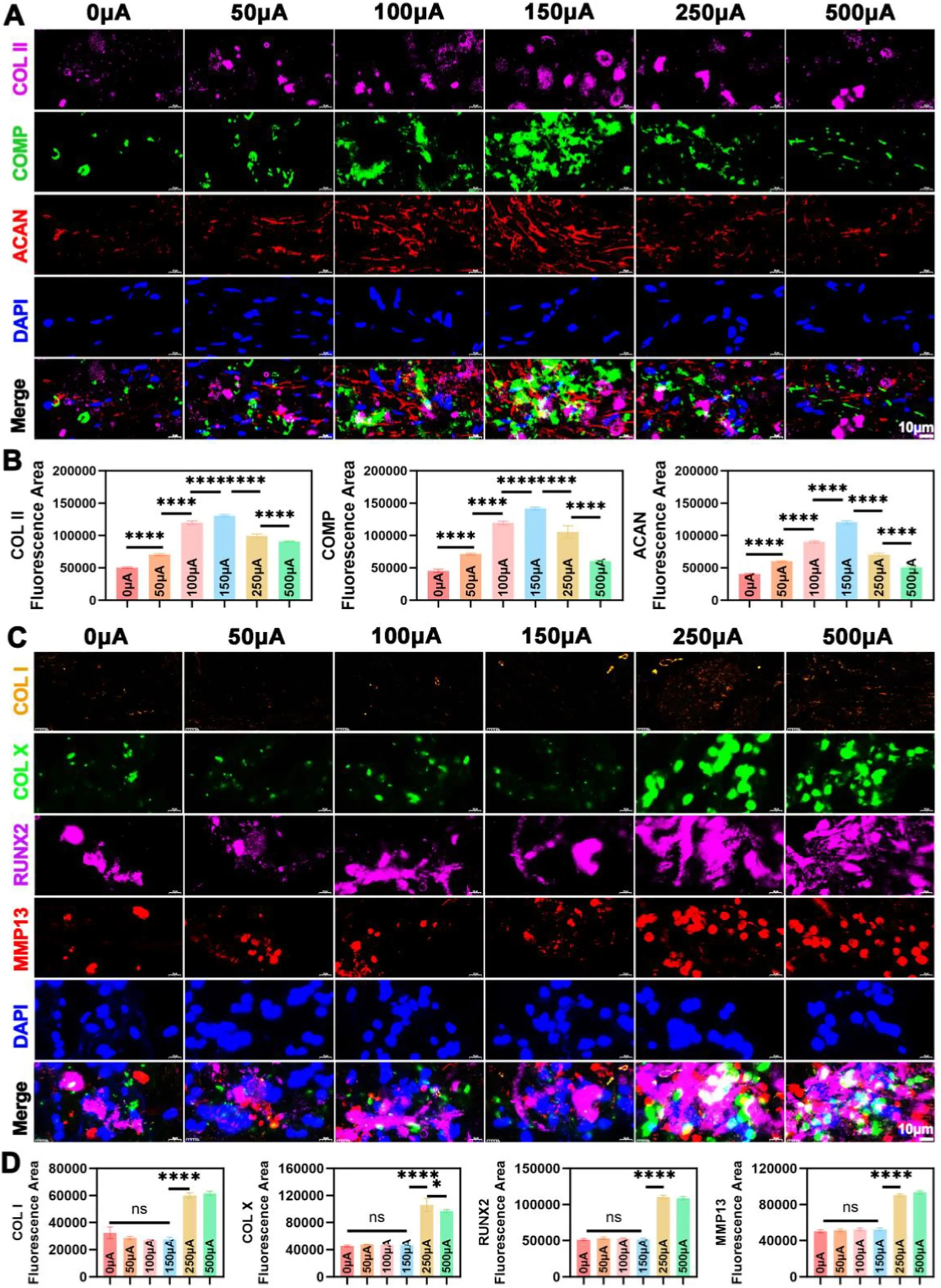
**Effects of different microcurrent stimulation intensities on chondrogenic ECM production and the expression of proteins associated with dedifferentiation, hypertrophy, and matrix degradation in BMSC-derived CMTs on day 14. (A)** Representative immunofluorescence staining images of COL II, COMP, and ACAN in BMSC-derived CMTs from the 0, 50, 100, 150, 250, and 500 μA groups. **(B)** Quantitative analysis of the fluorescence-positive areas of COL II, COMP, and ACAN based on the immunofluorescence staining shown in **(A)**. **(C)** Representative immunofluorescence images of COL I, COL X (the protein product encoded by Col10a1), RUNX2, and MMP13 in day-14 BMSC-derived CMTs exposed to 0, 50, 100, 150, 250, or 500 μA microcurrent stimulation. **(D)** Quantitative analysis of the fluorescence-positive areas of COL I, COL X, RUNX2, and MMP13 based on the immunofluorescence staining shown in **(C)**. Data are presented as mean ± SD (n = 15 independent rabbit donors). Field-level measurements were averaged to produce a single donor-level value for each marker. Multiple CMTs, sections, or microscopic fields obtained from the same donor-condition were treated as technical subsamples and averaged to generate a single donor-level value before statistical analysis. ns, not significant; *P < 0.05; ****P < 0.0001.

In contrast, COL I, COL X, RUNX2, and MMP13 staining revealed an opposite intensity-dependent pattern (Fig. 4C and D). COL I is associated with fibrocartilaginous or dedifferentiated matrix formation [30], COL X and RUNX2 are linked to hypertrophic differentiation [31], and MMP13 is a key matrix-degrading enzyme [32]. These unfavorable markers remained relatively low in the 0-150 μA groups, particularly under the 150 μA condition, but increased progressively in the 250 and 500 μA groups. Thus, the present data indicate that the 150 μA condition was associated with a more favorable cartilage-associated molecular phenotype, whereas excessive stimulation weakened cartilage-specific matrix formation and enhanced markers related to fibrotic deviation, hypertrophic maturation, and matrix degradation.

These findings are biologically relevant because BMSC chondrogenesis can be accompanied by hypertrophic differentiation and matrix remodeling [31]. Therefore, enhancement of chondrogenic markers alone does not necessarily indicate a favorable cartilage-like phenotype; concurrent assessment of fibrotic, hypertrophic, and catabolic features is also important.

To provide an independent quantitative molecular assessment of CMT phenotype, we performed RT-qPCR analysis of representative chondrogenic, fibrotic/hypertrophic, and catabolic genes at day 14 (Fig. S8). The chondrogenic genes *Col2a1*, *Sox9*, and *Acan* showed an intensity-dependent pattern, with the highest expression observed under the 150 μA condition and lower expression at higher stimulation intensities, particularly 500 μA (Fig. S8A). In contrast, the fibrotic/hypertrophic genes *Col1a1*, *Col10a1*, and *Runx2* remained comparatively low at 150 μA but increased under higher-current stimulation (Fig. S8B). A similar pattern was observed for the catabolic genes *Mmp13* and *Adamts5*, which were relatively low at 150 μA and increased particularly at 500 μA (Fig. S8C).

These transcriptional patterns were broadly consistent with the immunofluorescence findings and further support an intensity-dependent shift in CMT phenotype. Overall, the 150 μA condition was associated with a more favorable molecular profile, characterized by increased cartilage-associated gene expression together with comparatively lower fibrotic, hypertrophic, and catabolic gene expression. In contrast, higher-current stimulation, particularly at 500 μA, was associated with a less favorable molecular phenotype.

### 3.5 High-current microcurrent stimulation was associated with catabolic, inflammatory, apoptosis-related, and metabolic alterations in BMSC-derived CMTs

To further characterize the unfavorable phenotype associated with high-current stimulation in BMSC-derived CMTs, we examined catabolic, inflammatory, apoptosis-related, metabolic, and proliferation-related responses. Immunofluorescence staining showed that ADAMTS5, iNOS, and COX-2 were maintained at relatively low levels in the 0-150 μA groups, whereas their immunoreactivity increased progressively in the 250 and 500 μA groups on day 14 (Fig. 5A and B). ADAMTS5 is a major aggrecan-degrading enzyme [33], while iNOS and COX-2 are associated with inflammatory stress and catabolic cartilage remodeling [34]. The increased immunoreactivity for these markers was consistent with a catabolic and stress-associated phenotype in the high-current groups. RT-qPCR analysis further showed that *Adamts5* expression was relatively low under the 150 μA condition but increased with higher stimulation intensity, reaching its highest level at 500 μA (Fig. S8C). *Mmp13* showed a similar current-dependent pattern. These transcriptional findings were consistent with the immunofluorescence results and further demonstrated a less favorable matrix-remodeling profile under high-current stimulation. However, these associations do not establish inflammatory or catabolic signaling as the primary or sole cause of the impaired matrix phenotype.

**Fig. 5:**
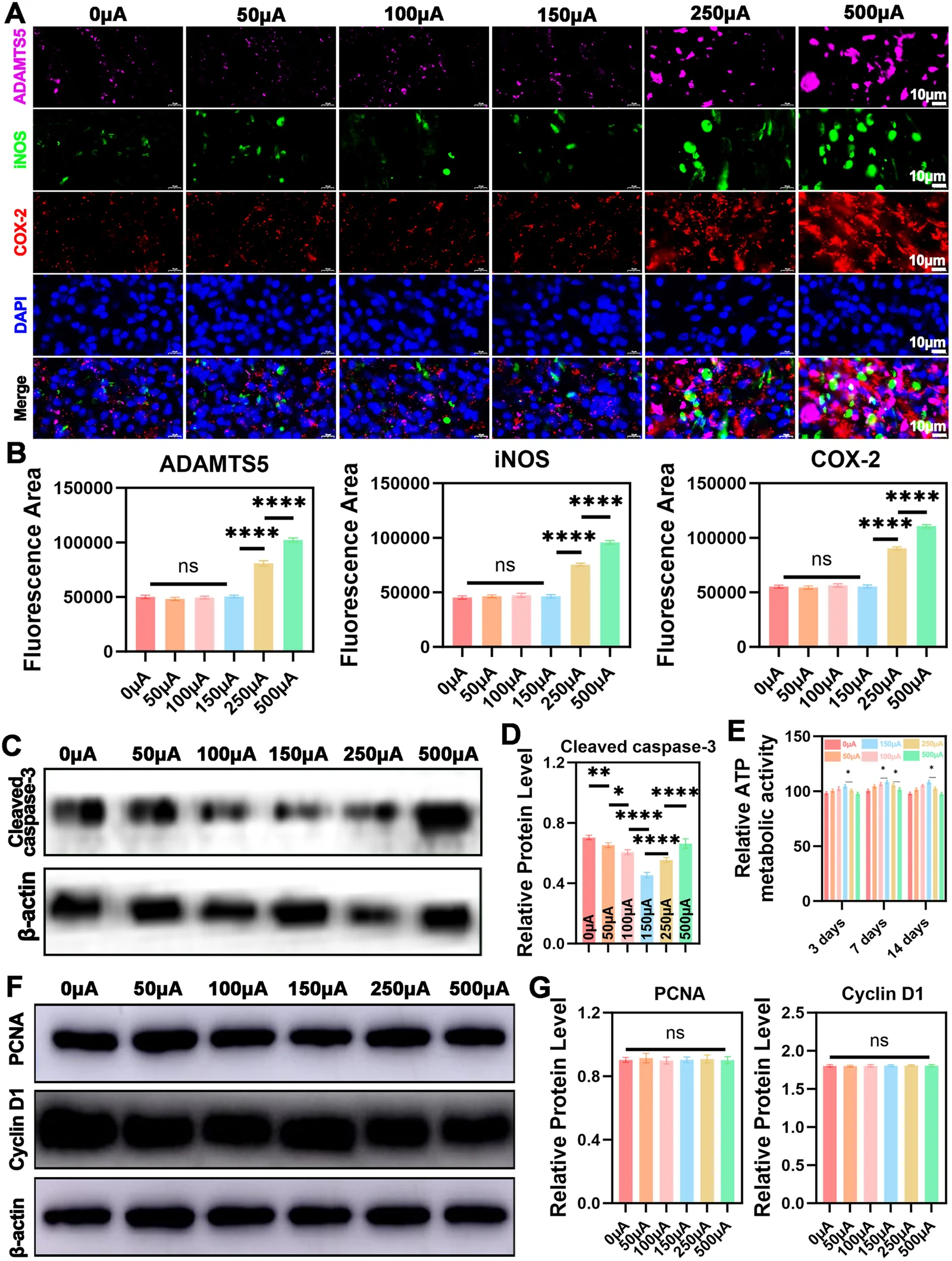
**Microcurrent stimulation regulated catabolic, inflammatory, apoptotic, metabolic, and proliferation-related responses in BMSC-derived CMTs. (A)** Representative immunofluorescence staining images of ADAMTS5, iNOS, and COX-2 in BMSC-derived CMTs from the 0, 50, 100, 150, 250, and 500 μA groups at day 14. **(B)** Quantitative analysis of the fluorescence-positive areas of ADAMTS5, iNOS, and COX-2 based on the immunofluorescence staining shown in **(A)**. **(C)** Representative Western blot images showing the protein expression level of cleaved caspase-3 in BMSC-derived CMTs from each group at day 14, with β-actin used as the loading control. **(D)** Quantitative analysis of the relative protein expression level of cleaved caspase-3 based on the Western blot results shown in **(C)**. (E) ATP-based metabolic activity of BMSC-derived CMTs from each group at days 3, 7, and 14, as measured by the CellTiter-Glo assay. **(F)** Representative Western blot images showing the protein expression levels of PCNA and Cyclin D1 in BMSC-derived CMTs from each group at day 14, with β-actin used as the loading control. **(G)** Quantitative analysis of the relative protein expression levels of PCNA and Cyclin D1 based on the Western blot results shown in **(F)**. Data are presented as mean ± SD (n = 15 independent rabbit donors; stimulation conditions matched within donors). Western blot and CellTiter-Glo measurements obtained from technical CMT samples from the same donor-condition were likewise averaged to generate one donor-level value. Technical CMTs, fields, and assay measurements were not treated as independent biological replicates. ns, not significant; *P < 0.05; **P < 0.01; ****P < 0.0001.

Live/Dead staining on days 3 and 7 showed no obvious differences in cell viability among the stimulation groups (Fig. S9), and TUNEL staining at the same time points revealed no significant differences in TUNEL-positive area (Fig. S10A–C). These findings indicate that high-current stimulation was not associated with widespread early cell death or a detectable increase in apoptosis during the initial phase of 3D culture. By day 14, however, apoptosis-related molecular changes became evident: cleaved caspase-3 and Bax were increased in the 250 and 500 μA groups, whereas Bcl-2 was decreased, resulting in an elevated Bax/Bcl-2 ratio (Fig. 5C, D and Fig. S11). Together, these findings are compatible with a delayed or cumulative stress-associated response during prolonged high-current exposure, with increased apoptosis-related signaling becoming apparent at later stages of CMT culture. However, the present data do not demonstrate immediate apoptosis after stimulation onset or establish apoptosis as the primary or sole cause of the unfavorable phenotype observed under high-current stimulation.

CellTiter-Glo analysis showed an intensity-dependent pattern of ATP-based metabolic activity across days 3, 7, and 14 (Fig. 5E), with higher activity observed from 0 to 150 μA and lower activity under 250 and 500 μA stimulation. Because the CellTiter-Glo assay reflects cellular ATP availability, these findings suggest that the 150 μA condition was associated with a more favorable metabolic state during CMT development, whereas higher-current stimulation was associated with reduced ATP-based metabolic activity. However, ATP measurements alone do not distinguish changes in cell number from alterations in cellular metabolic state and should therefore be interpreted together with the proliferation and viability data.

Western blotting for PCNA and Cyclin D1 (Fig. 5F and G), together with RT-qPCR analysis of *Ccnd1*, *Mki67*, and *Pcna* (Fig. S12), revealed no significant differences in proliferation-related markers among stimulation groups in the 3D CMT system. This differed from the reduced EdU incorporation and Ki67-positive rate observed under 250 and 500 μA stimulation in 2D monolayer culture. These findings are not necessarily contradictory, because the assays were performed at different time points, assessed non-identical proliferation-related endpoints, and were conducted in distinct culture contexts. Microcurrent responsiveness may differ between 2D and 3D systems owing to differences in cell density, cell–cell and cell–matrix interactions, ECM accumulation, and differentiation state [35]. Thus, the present data support reduced proliferative activity under high-current stimulation in the 2D system but do not demonstrate persistent suppression of proliferation in 3D CMTs.

The unfavorable phenotype observed under high-current stimulation should not be attributed to a single stress pathway. The present data suggest that excessive stimulation may involve both cellular stress responses and features of the local stimulation environment, although their relative contributions cannot be resolved by the current experiments.

### 3.6 Microcurrent stimulation was associated with intensity-dependent Ca^2+^ responses and NFATc1 nuclear localization in BMSC-derived CMTs

Because the Ca^2+^/calcineurin/NFAT axis was selected as a literature-supported candidate pathway for bioelectrical sensing, we examined whether microcurrent stimulation was associated with changes in intracellular Ca^2+^ dynamics and NFATc1 localization. The 0, 150, and 500 μA conditions were selected for focused signaling analysis to represent the unstimulated control, the best-performing condition identified in the exploratory intensity screen, and a high-current condition associated with impaired CMT quality, respectively.

Fluo-4 AM imaging of dissociated CMT-derived cells revealed distinct Ca^2+^ responses among the three conditions (Fig. 6A). Quantitative analysis showed that the area under the Ca^2+^ signal curve (AUC) increased from 0 to 500 μA during the first 60 s after stimulation onset (Fig. 6B). In contrast, the proportion of Ca^2+^-responsive cells was highest under the 150 μA condition and was lower at 500 μA (Fig. 6C). Thus, the high-current condition was characterized by a greater overall Ca^2+^ signal but a lower fraction of responsive cells than the 150 μA condition, indicating a distinct population-level Ca^2+^ response pattern. These findings establish an association between stimulation intensity and intracellular Ca^2+^ dynamics but do not demonstrate that altered Ca^2+^ signaling is the cause of the unfavorable high-current phenotype.

**Fig. 6:**
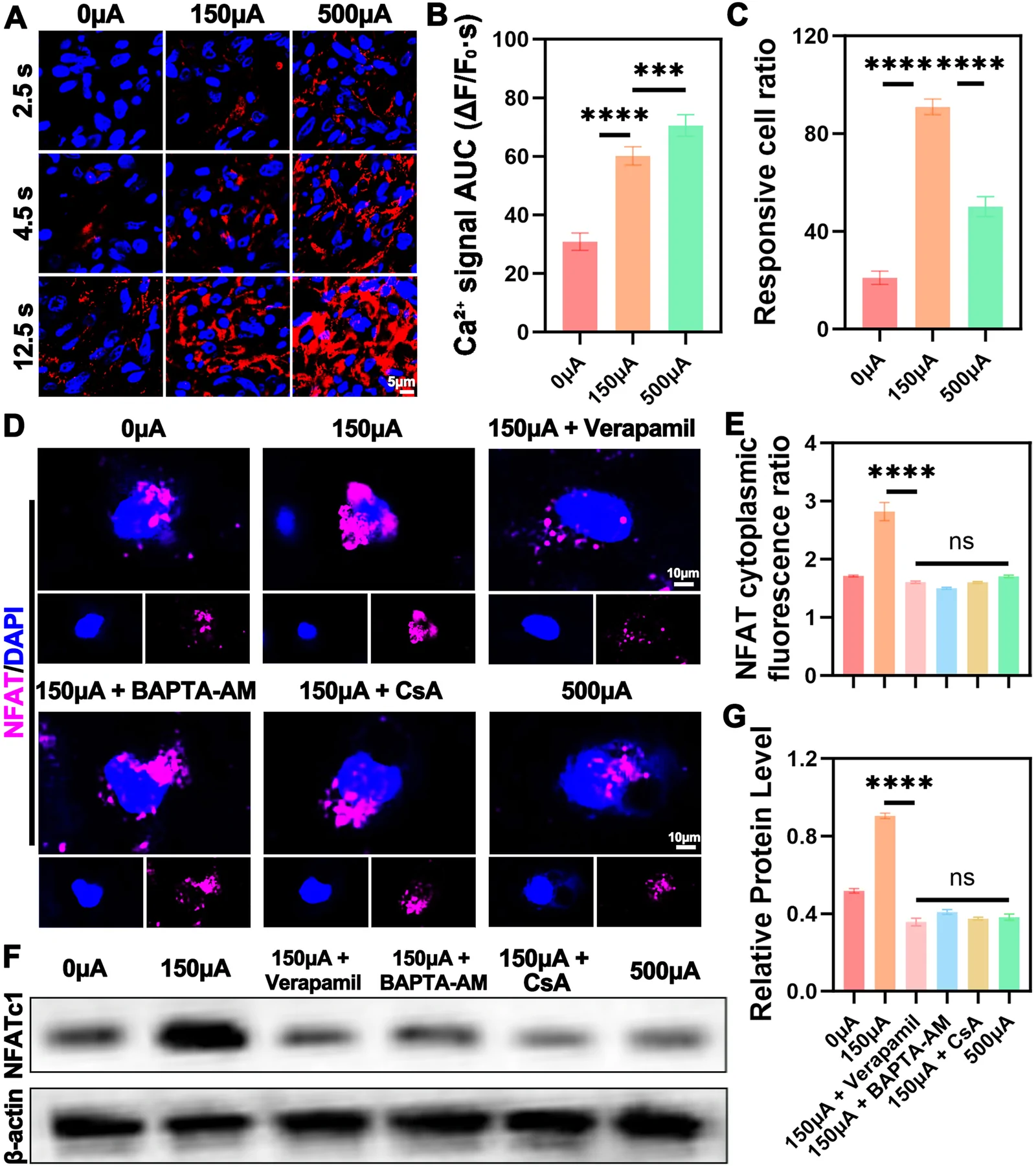
**Microcurrent stimulation regulated intracellular Ca^2+^ dynamics, NFATc1 nuclear localization, and total NFATc1 abundance in BMSC-derived CMTs. (A)** Representative Fluo-4 AM calcium imaging snapshots of BMSC-derived CMTs in the 0, 150, and 500 μA groups, captured at their respective peak-response time points of 2.5, 4.5, and 12.5 s. **(B, C)** Quantitative analysis of the Ca^2+^ signal area under the curve (AUC; ΔF/F_0_·s) and the responsive cell ratio in the 0, 150, and 500 μA groups during the first 60 s after stimulation onset. **(D)** Representative immunofluorescence staining images of NFAT and DAPI in BMSC-derived CMTs from the 0 μA, 150 μA, 150 μA + verapamil, 150 μA + BAPTA-AM, 150 μA + CsA, and 500 μA groups at day 14. **(E)** Quantitative analysis of the NFAT nuclear/cytoplasmic fluorescence ratio in each group. **(F)** Representative Western blot images showing total NFATc1 protein abundance in whole-cell lysates from the indicated groups, with β-actin as the loading control. **(G)** Quantitative analysis of total NFATc1 protein abundance based on the Western blot results shown in **(F)**. The pharmacological experiments were used to explore the potential involvement of Ca^2+^/calcineurin-associated signaling in NFATc1 nuclear localization. Because a matched DMSO vehicle-control group and unstimulated inhibitor-only controls were not included, these experiments were not intended to establish pathway-specific causality or causal mediation of downstream cartilage-matrix formation. For Ca^2+^ imaging, multiple dissociated CMT-derived cells were tracked within each imaging field; individual cells were treated as subsamples rather than independent biological replicates. Measurements were averaged hierarchically across cells, fields, and CMT-derived samples to generate a single donor-level value per condition. Data are presented as mean ± SD (n = 15 independent rabbit donors). All image analyses were performed blinded to group allocation. ns, not significant; ***P < 0.001; ****P < 0.0001.

We next examined NFATc1 as a Ca^2+^-responsive transcriptional regulator. The 150 μA condition was associated with increased NFATc1 nuclear localization, as indicated by both the nuclear/cytoplasmic fluorescence ratio and the proportion of cells exhibiting predominant nuclear NFATc1 localization (Fig. 6D and E; Fig. S13). Because the inhibitor-containing groups contained 0.1% DMSO and a matched DMSO vehicle-control group was not included, the following pharmacological comparisons should be interpreted cautiously, as potential vehicle-related or nonspecific treatment effects cannot be fully excluded. Verapamil, BAPTA-AM, and CsA attenuated NFATc1 nuclear localization relative to the 150 μA condition. The observed attenuation is therefore compatible with the involvement of Ca^2+^/calcineurin-associated signaling in NFATc1 nuclear localization but does not independently establish pathway causality.

Western blotting of whole-cell lysates further showed differences in total NFATc1 protein abundance among stimulation conditions (Fig. 6F and G). Because whole-cell lysates were analyzed, these data reflect total NFATc1 abundance rather than NFATc1 activation, phosphorylation state, or nuclear translocation. Similarly, although day-14 RT-qPCR showed that the 150 μA condition was associated with higher expression of the cartilage-related genes *Sox9*, *Col2a1*, and *Acan* (Fig. S8A), these genes cannot be regarded as direct NFAT targets on the basis of the present experiments because their expression was not examined under NFAT-specific genetic perturbation.

Overall, the Ca^2+^ imaging and pharmacological-interference results support the involvement of Ca^2+^/calcineurin/NFAT-related signaling in the cellular response to the 150 μA condition. However, nuclear localization alone does not establish NFAT transcriptional activity, and the present experiments do not demonstrate that NFATc1 directly mediates the downstream cartilage-matrix phenotype. Moreover, NFATc1 was examined as a representative calcineurin-responsive NFAT family member; other NFAT isoforms may also contribute. Therefore, the present findings should be interpreted as evidence of pathway involvement rather than as proof of a unique or complete causal mechanism.

### 3.7 CMTs preconditioned at 150 μA showed a more favorable short-term ectopic cartilage-like tissue phenotype after subcutaneous implantation

A schematic summary of the observed intensity-dependent responses and the proposed signaling relationships is shown in Fig. 7A. To determine whether differences established during *in vitro* microcurrent preconditioning were retained after transfer to an *in vivo* environment, rabbit BMSC-derived CMTs preconditioned at 0, 150, or 500 μA were implanted subcutaneously into nude mice for 2 weeks (Fig. 7B). These groups represented the unstimulated control, the best-performing tested condition, and a high-current condition associated with impaired CMT quality, respectively. The model was used as a short-term xenogeneic ectopic tissue-formation assay, rather than as a model of orthotopic cartilage-defect repair.

**Fig. 7:**
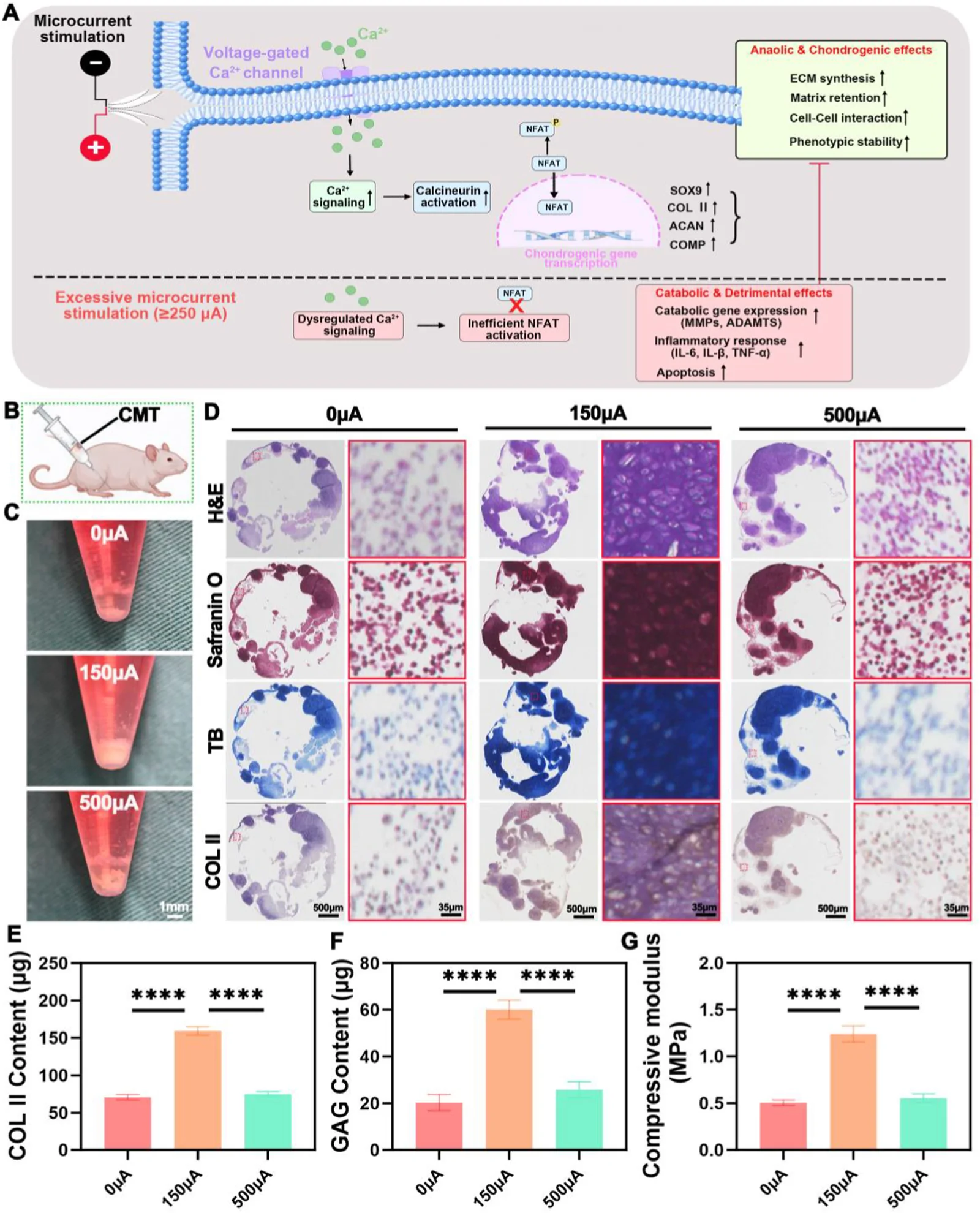
**Signaling-associated analysis and short-term xenogeneic ectopic implantation of microcurrent-preconditioned BMSC-derived CMTs. (A)** The Ca^2+^/calcineurin/NFAT axis is shown as a proposed signaling association supported by calcium imaging, NFATc1 nuclear-localization analysis, and pharmacological interference. Dashed arrows indicate relationships that were not established as definitive causal pathways in the present study. **(B)** Schematic illustration of the subcutaneous injection of BMSC-derived CMTs into nude mice. **(C)** Representative gross images of ectopically formed tissue samples from the 0, 150, and 500 μA groups after 2 weeks of subcutaneous implantation in nude mice. **(D)** Representative H&E, Safranin O, TB, and immunohistochemical COL II staining images of ectopically formed tissue samples from the 0, 150, and 500 μA groups after 2 weeks of subcutaneous implantation. The right panels show enlarged views of the corresponding regions indicated in the left panels. **(E–G)** Quantitative analysis of COL II content, GAG content, and compressive modulus of ectopically formed tissue samples from the 0, 150, and 500 μA groups after 2 weeks of subcutaneous implantation. Data are presented as mean ± SD (n = 5 independent mice per group; one implantation site per mouse). The individual mouse was the biological and statistical experimental unit. Multiple histological sections, microscopic fields, and analytical measurements from the same explanted tissue were treated as subsamples and were not considered independent biological replicates. ****P < 0.0001.

Gross examination showed clear differences among the explants, with the 150 μA-preconditioned group exhibiting more substantial tissue formation than the 0 and 500 μA groups (Fig. 7C). Histological analysis further demonstrated a more organized tissue structure and greater cartilage-like matrix accumulation in the 150 μA group. Safranin O and Toluidine Blue staining showed stronger proteoglycan-rich matrix deposition, while COL II immunohistochemistry showed greater COL II immunoreactivity than in the 0 or 500 μA groups (Fig. 7D). Quantitative biochemical and mechanical measurements were consistent with the histological findings. Native COL II content, GAG content, and apparent compressive modulus were higher in the 150 μA-preconditioned group than in the other tested conditions (Fig. 7E–G). These measurements characterize the explanted tissue as a whole and do not distinguish matrix originating from persistent rabbit donor cells from matrix associated with host-mediated remodeling.

Together, these findings indicate that CMTs preconditioned at 150 μA were associated with a more favorable short-term ectopic cartilage-like tissue phenotype, characterized by greater cartilage-like matrix accumulation and improved apparent compressive properties after 2 weeks of subcutaneous implantation. These results support persistence of differences associated with *in vitro* preconditioning after implantation, but they should not be interpreted as evidence of stable articular cartilage regeneration or cartilage-defect repair.

### 3.8 Limitations

Several limitations should be considered when interpreting the present findings. First, the identified stimulation response is specific to the electrical configuration used in this study, including the electrode geometry, exposed electrode area, inter-electrode distance, medium volume, sample position, and continuous unidirectional DC protocol. The nominal current should therefore not be regarded as a universally transferable biological dose. Moreover, the intensity screen was exploratory: no single primary optimization endpoint or formal dose-selection rule was prospectively specified, and 150 μA was selected as the best-performing tested condition based on convergence of molecular, biochemical, morphological, and mechanical outcomes within the same dataset. An independent confirmatory dose-selection cohort was not included. Accordingly, future studies should prospectively define a primary endpoint and independently validate the selected condition against neighboring intensities.

Second, continuous unidirectional DC stimulation may produce electrode-interface and electrochemical effects that were not fully characterized. Although no detectable changes in bulk medium temperature, bulk pH, or the measured cellular ROS signal were observed under the tested conditions, localized pH or redox gradients, electrode polarization, dissolved-gas changes, medium conductivity or osmolality, platinum dissolution, and other cumulative electrode-associated effects were not systematically quantified. The biological responses observed here should therefore be interpreted as responses to the overall electrical stimulation environment, rather than as evidence of an exclusively bioelectrical mechanism.

Third, the temporal window during which microcurrent exerted its favorable effect could not be resolved. Stimulation was applied continuously throughout chondrogenic induction, whereas the principal cartilage-matrix endpoints were assessed at day 14. Although early viability and apoptosis-related measurements were obtained on days 3 and 7, the present design cannot distinguish effects on early lineage commitment from effects on later extracellular-matrix accumulation. Temporally restricted stimulation, delayed-start, withdrawal, and longer longitudinal experiments will be required to determine whether the response depends predominantly on early, late, or sustained electrical exposure.

Fourth, the mechanistic experiments support pathway involvement but do not establish a complete causal mechanism. The Ca^2+^/calcineurin/NFAT axis was investigated on a hypothesis-driven basis rather than being identified through unbiased pathway screening. Pharmacological treatment with verapamil, BAPTA-AM, and CsA was associated with attenuation of NFATc1 nuclear localization under 150 μA stimulation. However, a matched DMSO vehicle-control group and unstimulated inhibitor-only groups were not included in the original experimental design. Therefore, potential vehicle-related or nonspecific pharmacological effects cannot be fully separated from pathway-specific effects. Moreover, these experiments did not test whether pharmacological interference rescued or suppressed the complete cartilage-matrix phenotype. Verapamil also does not identify a specific Ca^2+^-entry pathway, and contributions from mechanosensitive channels, purinergic signaling, or intracellular Ca^2+^ stores cannot be excluded. Furthermore, NFATc1 nuclear localization does not directly measure transcriptional activity, whole-cell Western blotting reflects total NFATc1 abundance, and NFAT phosphorylation, nuclear/cytoplasmic biochemical fractionation, transcriptional reporter activity, direct NFAT binding to target genes, and other NFAT isoforms were not examined. Thus, the current data support Ca^2+^/calcineurin/NFAT-related signaling involvement, but not NFATc1-specific or exclusive causal mediation.

Fifth, the RT-qPCR analysis has methodological limitations. Primer-specific amplification efficiencies were not experimentally determined using serial-dilution standard curves, and Gapdh was the only reference gene used. Although re-examination of raw Gapdh Cq values showed no systematic current-dependent shift, multiple candidate reference genes were not prospectively evaluated. The assumptions underlying the 2^−ΔΔCt^ analysis should therefore be considered when interpreting the reported fold changes.

Finally, the *in vivo* experiment was a short-term xenogeneic ectopic proof-of-concept model. Rabbit BMSC-derived CMTs were implanted into immunodeficient nude mice, donor cells were not lineage-traced, and species-specific analyses were not performed; consequently, donor- and host-derived contributions to the explanted tissue cannot be resolved. Host inflammatory and vascular responses were also not systematically assessed. In addition, the 2-week implantation period was insufficient to determine long-term phenotypic stability or exclude subsequent hypertrophy, fibrosis, vascular invasion, mineralization, calcification, or endochondral ossification. The subcutaneous site does not reproduce physiological joint loading, synovial exposure, the osteochondral interface, or integration with native cartilage. Future studies should therefore incorporate longer-term, lineage-resolved and species-matched experiments, followed by orthotopic cartilage-defect models assessing tissue integration, hypertrophic/mineralization stability, repair-tissue mechanics, and functional outcomes.

## 4 Conclusion

In this study, continuous microcurrent stimulation regulated BMSC-derived CMT formation in an intensity-dependent manner under the defined electrical and culture configuration. Among the tested intensities, 150 μA produced the most consistently favorable overall response, characterized by enhanced cartilage-associated matrix deposition, improved CMT morphology, and greater apparent mechanical properties. In contrast, higher stimulation intensities, particularly 250 and 500 μA, were associated with less favorable matrix, stress-related, catabolic, hypertrophic, and late-stage apoptosis-related phenotypes. The 150 μA condition was also associated with distinct intracellular Ca^2+^ responses and increased NFATc1 nuclear localization, supporting the involvement of Ca^2+^/calcineurin/NFAT-related signaling without establishing NFATc1 as a unique or causal mediator of the cartilage-like phenotype.

After 2 weeks of xenogeneic subcutaneous implantation in nude mice, CMTs preconditioned at 150 μA exhibited greater cartilage-like matrix accumulation and higher apparent compressive properties than those preconditioned at 0 or 500 μA. These findings provide short-term ectopic proof-of-concept evidence that differences associated with 150 μA preconditioning remained detectable after implantation, but they do not establish long-term cartilage stability, articular cartilage-defect repair, or regenerative efficacy. Further studies using longer-term, lineage-resolved, orthotopic, and immunocompetent models, together with assessment of hypertrophic/mineralization stability, host responses, tissue integration, and performance under physiological loading, will be required to establish the translational potential of microcurrent-preconditioned CMTs.

## CRediT authorship contribution statement

**Xiang Fei:** Data curation, Conceptualization. **Hao Wu:** Funding acquisition, Formal analysis. **Mengxing Li:** Methodology, Investigation. **Yong Xu:** Supervision, Software. **Hongtao He:** Resources, Project administration. **Zhao An:** Writing – review & editing, Writing – original draft, Visualization, Validation.

## Declaration of competing interest

The authors declare no conflict of interest.

## Data Availability

Data will be made available on request.
