# Supplementary material for: Intensity-dependent microcurrent stimulation enhances BMSC-derived cartilage microtissue formation under defined culture conditions
Source: Mater Today Bio. 2026 Sep 19;41:103698. doi: 10.1016/j.mtbio.2026.103698 (PMC13631619; doi:10.1016/j.mtbio.2026.103698)
Supplement: Supplementary file 1 [file mmc1.docx]

**Supporting Information**

**Intensity-Dependent Microcurrent Stimulation Enhances BMSC-Derived Cartilage Microtissue Formation under Defined Culture Conditions**

*Xiang Fei^1†^, Hao Wu^2†^, Mengxing Li^1†^, Yong Xu^1*^, Hongtao He^3*^, Zhao An^1*^*

^1^ Department of Thoracic Surgery, Shanghai Pulmonary Hospital, School of Medicine, Tongji University, Shanghai, China;

^2^ Department of Clinical Laboratory, Shanghai Municipal Hospital of Traditional Chinese Medicine, Shanghai University of Traditional Chinese Medicine, Shanghai, China;

^3^ Department of Orthopedics, the Second Hospital of Dalian Medical University, Dalian, China.

^†^ These authors contributed equally.

* Corresponding authors. E-mail address: Dr. Yong Xu ([xuyong@tongji.edu.cn](mailto:xuyong@tongji.edu.cn)); Dr. Hongtao He (hht359718376@163.com); Dr. Zhao An (anzhao1@yeah.net).

**Figure S1. Schematic illustration of the microcurrent stimulation system used for BMSC/CMT culture.** Schematic illustration of the constant-current stimulation setup, showing the platinum electrodes, exposed electrode area (10 mm × 5 mm), electrode spacing (15 mm), medium volume (2 mL), and the position of cells/CMTs in the central culture region between the electrodes.

**Figure S2. Engineering workflow for fabrication and microcurrent conditioning of BMSC-derived CMTs.** Schematic workflow of BMSC-derived CMT fabrication. Passage-3 rabbit BMSCs were resuspended in chondrogenic induction medium and seeded at 1 × 10^5^ cells per 1000-μm round-bottom microwell. Following three-dimensional aggregation, CMTs were maintained under chondrogenic induction with continuous direct-current microcurrent stimulation (24 h/day, except during medium replacement). Early viability and apoptosis were assessed on days 3 and 7, and major construct-level analyses were performed at day 14. Electrical-system parameters are detailed separately in Section 2.2 and the corresponding stimulation-system schematic.

**Figure S3.** CCK-8 analysis of BMSCs exposed to microcurrent stimulation at 0, 50, 100, 150, 250, and 500 μA during 2D chondrogenic induction on days 0, 1, 3, 5, 9, and 11. **Data are presented as mean ± SD (n = 15 independent rabbit donors; stimulation conditions matched within donors). Technical culture wells from the same donor-condition were averaged before donor-level statistical analysis.**

**Figure S4. Microcurrent stimulation did not substantially alter the culture microenvironment or induce ROS accumulation during 2D chondrogenic induction.** **(A)** Temperature and **(B)** pH of the 2D culture medium after microcurrent stimulation at 0, 50, 100, 150, 250, and 500 μA on days 1, 5, and 9. **(C)** ROS levels after microcurrent stimulation at 0, 50, 100, 150, 250, and 500 μA during 2D chondrogenic induction on day 5. Data are presented as mean ± SD (n = 15 independent rabbit donors; stimulation conditions matched within donors). Technical culture wells from the same donor-condition were averaged before donor-level statistical analysis. ns, not significant.

**Figure S5.** Quantitative analysis of the shrinkage rate of BMSC-derived CMTs exposed to microcurrent stimulation at 0, 50, 100, 150, 250, and 500 μA on day 14. **Data are presented as mean ± SD (n = 15 independent rabbit donors). Multiple CMTs analyzed from the same donor-condition were treated as technical subsamples and averaged to generate a single donor-level shrinkage value before statistical analysis. *****P* < 0.0001.**

**Figure S6. Mechanical properties and hydration status of BMSC-derived CMTs under different microcurrent stimulation intensities on day 14.** **(A)** Compressive modulus and **(B)** dynamic storage modulus of BMSC-derived CMTs in the 0, 50, 100, 150, 250, and 500 μA groups. **(C)** Water content and **(D)** wet-to-dry weight ratio of BMSC-derived CMTs in the indicated groups. Data are presented as mean ± SD (n = 15 independent rabbit donors). Mechanical and hydration measurements obtained from multiple CMTs from the same donor-condition were averaged before donor-level analysis. ns, not significant; **P* < 0.05; ***P* < 0.01; ****P* < 0.001; *****P* < 0.0001.

**Figure S7.** Enlarged H&E and Safranin O images showing detailed cartilage-like matrix organization of BMSC-derived CMTs at day 14 in 0, 50, 100, 150, 250, 500 μA groups.

**Figure S8. RT-qPCR characterization of chondrogenic, fibrotic/hypertrophic, and catabolic markers in BMSC-derived CMTs at day 14. BMSC-derived CMTs were cultured under continuous microcurrent stimulation at 0, 50, 100, 150, 250, or 500 μA for 14 days. (A) Relative mRNA expression of the chondrogenic markers *Col2a1*, *Sox9*, and *Acan*. (B) Relative mRNA expression of the fibrotic/hypertrophic markers *Col1a1*, *Col10a1*, and *Runx2*. (C) Relative mRNA expression of the catabolic matrix-remodeling markers *Mmp13* and *Adamts5*. Gene expression was normalized to Gapdh and expressed relative to the 0 μA group using the 2^−ΔΔCt^ method. Data are presented as mean ± SD (n = 15 independent rabbit donors). ns, not significant; **P* < 0.05; ***P* < 0.01; ****P* < 0.001; *****P* < 0.0001.**

**Figure S9.** Representative Live/Dead staining images of BMSC-derived CMTs at day 3 and 7 in 0, 50, 100, 150, 250, 500 μA groups.

**Figure S10. Microcurrent stimulation did not markedly induce apoptosis in BMSC-derived CMTs during early 3D chondrogenic culture. (A)** Representative TUNEL staining images of BMSC-derived CMTs exposed to microcurrent stimulation at 0, 50, 100, 150, 250, and 500 μA on days 3 and 7. **(B, C)** Quantitative analysis of the TUNEL-positive fluorescence area in the indicated groups on days 3 and 7. Data are presented as mean ± SD (n = 15 independent rabbit donors). Multiple CMTs, sections, and microscopic fields from the same donor-condition were treated as technical subsamples and averaged before statistical analysis. ns, not significant**.**

**Figure S11. Microcurrent stimulation modulated apoptosis-related protein expression in BMSC-derived CMTs on day 14. (A)** Representative western blot images of Bax and Bcl-2 protein expression in BMSC-derived CMTs exposed to microcurrent stimulation at 0, 50, 100, 150, 250, and 500 μA on day 14. **(B, C)** Quantitative analysis of relative Bax and Bcl-2 protein levels in the indicated groups. **(D)** Quantitative analysis of the relative Bax/Bcl-2 ratio in the indicated groups. Data are presented as mean ± SD (n = 15 independent rabbit donors; stimulation conditions were matched within donors). CMTs and repeated measurements originating from the same donor under the same stimulation condition were treated as technical subsamples and were summarized to generate a single donor-level value before statistical analysis. Individual CMTs or repeated Western blot measurements were not treated as independent biological replicates. ns, not significant; *****P* < 0.0001**.**

**Figure S12.** Relative mRNA expression levels of proliferation-related genes (*Ccnd1*, *Mki67*, and *Pcna*) of BMSC-derived CMTs at day 3, 7, and 14 in 0, 50, 100, 150, 250, 500 μA groups. Data are presented as mean ± SD (n = 15 independent rabbit donors). Donor identity was maintained across stimulation conditions and experimental time points. Technical culture and RT-qPCR measurements obtained from the same donor under the same stimulation condition and at the same time point were averaged to generate a single donor-level value. Technical wells and repeated RT-qPCR measurements were not treated as independent biological replicates. Statistical analysis was performed using a mixed-effects model with stimulation intensity and time as fixed effects and donor as a random effect, followed by Tukey’s multiple-comparisons test. ns, not significant.

**Figure S13.** Quantitative analysis of cells showing nuclear NFATc1 localization in the 0 μA, 150 μA, 150 μA + verapamil, 150 μA + BAPTA-AM, 150 μA + CsA, and 500 μA groups. The final DMSO concentration was 0.1% (v/v) in all inhibitor-containing groups; no matched DMSO vehicle-control group was included. Data are presented as mean ± SD (n = 15 independent rabbit donors; mechanistic treatment conditions were matched within donors). Multiple cells, microscopic fields, and CMTs analyzed from the same donor under the same treatment condition were considered subsamples and were averaged hierarchically to generate a single donor-level value before statistical analysis. Individual cells, fields, or CMTs were not treated as independent biological replicates. ns, not significant; **P* < 0.05; *****P* < 0.0001**.**

**Table S1. Electrical stimulation parameters used for BMSC-derived CMT culture.**

| **Parameter** | **Value** |
| --- | --- |
| Current source | Keithley 6221 |
| Stimulation mode | Continuous unidirectional direct current |
| Applied currents | 0, 50, 100, 150, 250, 500 μA |
| Pt electrode exposed dimensions | 10 × 5 mm |
| Pt electrode exposed area | 50 mm^2^ |
| Inter-electrode distance | 15 mm |
| Culture-medium volume | 2 mL |
| Nominal current densities | 0, 1, 2, 3, 5, 10 μA/mm^2^ |
| Equivalent current densities | 0, 100, 200, 300, 500, 1000 μA/cm^2^ |
| Voltage measured at 150 μA | ~0.3 V |
| Estimated bulk-average field at 150 μA | ~20 V/m (0.2 V/cm) |
| Stimulation duration | 24 h/day throughout induction, except medium replacement |

**Table S2. Primer sequences used for RT-qPCR analysis of rabbit (*Oryctolagus cuniculus*) samples.**

| **Genes** | **Forward primer (5′-3′)** | **Reverse primer (5′-3′)** |
| --- | --- | --- |
| *Col2a1* | CACGCTCAAGTCCCTCAACA | TCTATCCAGTAGTCACCGCTCT |
| *Sox9* | GCGTCAACGGCTCCAGCAAGA | GCGTTGTGCAGGTGCGGGTAC |
| *Acan* | CAGGCTATGAGCAGTGTGATGC | GCTGCTGTCTTTGTCACCCACA |
| *Ccnd1* | ATGCTCAAGGCGGAGGAGACCT | ACCTCCTCCTCGCACTTCTGCT |
| *Mki67* | TCCCTGAGGGCAATGGTTCAGG | ACGATGTCCAGGATGGGCTGAG |
| *Pcna* | GTCCTGAGCCTCGCGAAGAAGT | CTTGAGTGCCTCCAGCACCTTC |
| *Col1a1* | GACATGTTCAGCTTTGTGGACCTC | GGGACCCTTAGGCCATTGTGTA |
| *Col10a1* | GGAGAGCCAGGGTTGCCAG | GTCCTCTCTCCCCTTGTTTTCC |
| *Runx2* | TCAGGCATGTCCCTCGGTAT | TGGCAGGTAGGTATGGTAGTGG |
| *Mmp13* | TGAGATCATACTACCATCCTCTGAATCC | CAAGTTTGCCTGTCACCTCTAAGC |
| *Adamts5* | CTTCCACTAAGTAGTCCATGTAGATTGC | GGTCATTCCGATGTGGATTGC |
| *Gapdh* | TTGTCGCCATCAATGATCCAT | GATGACCAGCTTCCCGTTCTC |

**Table S3. Primary antibodies used in rabbit-derived samples and manufacturer-reported species-reactivity information.**

| **Target** | **Catalog no.** | **Host species** | **Application in this study** | **Manufacturer-reported species reactivity** | **Expected MW** |
| --- | --- | --- | --- | --- | --- |
| COL II | Abcam ab34712 | Rabbit polyclonal | IHC | Human, cow | 97 kDa* |
| COL II | Proteintech 28459-1-AP | Rabbit polyclonal | IF, WB | Tested: human; cited: human, mouse, rat, pig, **rabbit/Oryctolagus cuniculus**, goat | Calculated 134 kDa; observed ~200 kDa |
| COMP | Proteintech 28369-1-AP | Rabbit polyclonal | IF | Tested: human, mouse, rat, pig | 83 kDa; observed 80–110 kDa |
| ACAN | Proteintech 13880-1-AP | Rabbit polyclonal | IF, WB | Tested: human, mouse, rat; cited includes **rabbit** | Calculated ~250 kDa |
| COL I/COL1A2 | Proteintech 14695-1-AP | Rabbit polyclonal | IF | Tested: human, pig; cited: human, mouse, pig, canine, chicken, bovine, hamster, goat | 130 kDa; observed 130–160 kDa |
| COL X/COL10A1 | Proteintech 26984-1-AP | Rabbit polyclonal | IF | Tested: human, mouse, rat; cited includes **rabbit** | 66 kDa; observed 60–66 kDa |
| RUNX2 | Proteintech 20700-1-AP | Rabbit polyclonal | IF | Tested: human, rat; cited includes **rabbit** | 57 kDa; observed 57–60 kDa |
| MMP13 | Proteintech 18165-1-AP | Rabbit polyclonal | IF | Tested: human, mouse, rat; cited includes **rabbit** | 54 kDa; observed 65–70, 50–55 and ~40 kDa |
| ADAMTS5 | Affinity Biosciences DF13268 | Rabbit polyclonal | IF | Tested: human, mouse, rat; **predicted rabbit reactivity, 100% sequence identity** | Calculated 102 kDa; observed ~72/100 kDa |
| iNOS/NOS2 | Proteintech 80517-1-RR | Rabbit recombinant monoclonal | IF | Tested: human, mouse, rat | 131 kDa; observed 110–130 kDa |
| COX-2/PTGS2 | Proteintech 12375-1-AP | Rabbit polyclonal | IF | Tested: human, mouse; cited: human, mouse, rat, pig, monkey, chicken | 68 kDa; observed 70–74 kDa |
| NFATc1/NFATC1 | Proteintech 30114-1-AP | Rabbit polyclonal | IF, WB | Tested: human; cited: mouse, rat | 78/101 kDa isoforms; observed 82–140 kDa |
| SOX9 | Proteintech 67439-1-Ig | Mouse monoclonal | WB | Tested: human, mouse; cited includes **rabbit** | 56 kDa |
| Cleaved caspase-3 (Asp175) | CST #9661 | Rabbit polyclonal | WB | Human, mouse, rat, monkey; predicted: bovine, dog, pig | 17/19 kDa |
| PCNA | Proteintech 10205-2-AP | Rabbit polyclonal | WB | Tested: human, mouse, rat | 29 kDa; modified form ~36 kDa |
| Cyclin D1 | Proteintech 26939-1-AP | Rabbit polyclonal | WB | Tested: human, mouse, rat | 34 kDa |
| Bax | Proteintech 50599-2-Ig | Rabbit polyclonal | WB | Tested: human, mouse, rat | ~21.1 kDa |
| Bcl-2 | Proteintech 12789-1-AP | Rabbit polyclonal | WB | Tested: human | 26 kDa; observed 25–30 kDa |
| β-actin | Proteintech 66009-1-Ig | Mouse monoclonal | WB | Tested: human, mouse, rat, pig, **rabbit**, canine, monkey, chicken, zebrafish, hamster | 42 kDa |

**Footnote:** Manufacturer-reported “tested reactivity” indicates species experimentally tested by the manufacturer. “Cited reactivity” indicates species reported in publications indexed by the manufacturer and should not be interpreted as equivalent to manufacturer in-house validation. “Predicted reactivity” is based on sequence homology and likewise does not constitute experimental validation. Expected molecular weights are provided primarily as reference information for Western blot analyses and do not independently validate antibody specificity in immunofluorescence or immunohistochemistry.
